# Using an intersectionality-based approach to evaluate mental health services use among gay, bisexual and other men who have sex with men in Montreal, Toronto and Vancouver

**DOI:** 10.1017/S2045796024000143

**Published:** 2024-03-05

**Authors:** Ivan Marbaniang, Erica E. M. Moodie, Eric Latimer, Shayna Skakoon-Sparling, Trevor A. Hart, Daniel Grace, David M. Moore, Nathan J. Lachowsky, Jody Jollimore, Gilles Lambert, Terri Zhang, Milada Dvorakova, Joseph Cox, Engage Team Members

**Affiliations:** 1 Department of Epidemiology, McGill University, Montreal, QC, Canada; 2 Department of Epidemiology, Biostatistics and Occupational Health, McGill University, Montreal, QC, Canada; 3 Mental Health and Society Division, Douglas Research Centre, Montreal, QC, Canada; 4 Department of Psychiatry, McGill University, Montreal, QC, Canada; 5 Department of Psychology, Toronto Metropolitan University, Toronto, ON, Canada; 6 Department of Psychology, University of Guelph, Guelph, ON, Canada; 7 Dalla Lana School of Public Health, University of Toronto, Toronto, ON, Canada; 8 British Columbia Centre for Excellence in HIV/AIDS, Vancouver, BC, Canada; 9 Department of Medicine, Division of Infectious Disease, University of British Columbia, Vancouver, BC, Canada; 10 School of Public Health and Social Policy, University of Victoria, Victoria, BC, Canada; 11 Community Based Research Centre, Vancouver, BC, Canada; 12 Institut National de Santé Publique du Québec, Montreal, QC, Canada; 13 Clinical Outcomes Research and Evaluation, Research Institute–McGill University Health Centre, Montreal, QC, Canada

**Keywords:** bisexual, Canada, discrimination, gay, intersectionality, mental health services, men who have sex with men

## Abstract

**Aims:**

To cope with homonegativity-generated stress, gay, bisexual and other men who have sex with men (GBM) use more mental health services (MHS) compared with heterosexual men. Most previous research on MHS among GBM uses data from largely white HIV-negative samples. Using an intersectionality-based approach, we evaluated the concomitant impact of racialization and HIV stigma on MHS use among GBM, through the mediating role of perceived discrimination (PD).

**Methods:**

We used baseline data from 2371 GBM enrolled in the Engage cohort study, collected between 2017 and 2019, in Montreal, Toronto and Vancouver, using respondent-driven sampling. The exposure was GBM groups: **Group 1** (*n* = 1376): white HIV-negative; **Group 2** (*n* = 327): white living with HIV; **Group 3** (*n* = 577): racialized as non-white HIV-negative; **Group 4** (*n* = 91): racialized as non-white living with HIV. The mediator was interpersonal PD scores measured using the Everyday Discrimination Scale (5-item version). The outcome was MHS use (yes/no) in the prior 6 months. We fit a three-way decomposition of causal mediation effects utilizing the imputation method for natural effect models. We obtained odds ratios (ORs) for pure direct effect (PDE, unmediated effect), pure indirect effect (PIE, mediated effect), mediated interaction effect (MIE, effect due to interaction between the exposure and mediator) and total effect (TE, overall effect). Analyses controlled for age, chronic mental health condition, Canadian citizenship, being cisgender and city of enrolment.

**Results:**

Mean PD scores were highest for racialized HIV-negative GBM (10.3, SD: 5.0) and lowest for white HIV-negative GBM (8.4, SD: 3.9). MHS use was highest in white GBM living with HIV (GBMHIV) (40.4%) and lowest in racialized HIV-negative GBM (26.9%). Compared with white HIV-negative GBM, white GBMHIV had higher TE (OR: 1.71; 95% CI: 1.27, 2.29) and PDE (OR: 1.68; 95% CI: 1.27, 2.24), and racialized HIV-negative GBM had higher PIE (OR: 1.09; 95% CI: 1.02, 1.17). Effects for racialized GBMHIV did not significantly differ from those of white HIV-negative GBM. MIEs across all groups were comparable.

**Conclusions:**

Higher MHS use was observed among white GBMHIV compared with white HIV-negative GBM. PD positively mediated MHS use only among racialized HIV-negative GBM. MHS may need to take into account the intersecting impact of homonegativity, racism and HIV stigma on the mental health of GBM.

## Introduction

Despite Canada’s considerable progress on sexual minority rights over the past two decades (Rau, 2021), discriminatory attitudes against gay, bisexual and other men who have sex with men (GBM) persist (Government of Canada, 2023c; Hyman *et al.*, 2019). Biopsychosocial models and minority stress theory conceptualize discrimination as a social stressor (Bogart *et al.*, 2011; Brondolo *et al.*, 2018; Meyer, 2003). Compared with heterosexual men, heightened stress in GBM, secondary to societal homonegativity, triggers maladaptive psychological pathways that increase the risk of depression, anxiety, substance use and suicidality (Hatzenbuehler, 2009). This may contribute to GBM using mental health services (MHS) more frequently relative to heterosexual men (Hart *et al.*, 2023; Marbaniang *et al.*, 2022; Platt *et al.*, 2018; Tjepkema, 2008), as a means to cope with negative psychological states that result from sexual minority stress (Giwa and Han, 2022).

Research on MHS use among GBM has typically relied upon data from predominantly white samples (Ferlatte *et al.*, 2017; Kulick, 2013). However, GBM in Canada are not a racially monolithic group (Government of Canada, 2023b). In addition to homonegativity, GBM racialized as non-white encounter racism both from mainstream society at large and other GBM (Choi *et al.*, 2013; Giwa and Han, 2022; Jackson *et al.*, 2020; McConnell *et al.*, 2018; Sadika *et al.*, 2020). Like homonegativity, racism negatively affects mental health (Brondolo *et al.*, 2009; Paradies *et al.*, 2015). In the general population, racialized individuals tend to underutilize MHS and, instead, are more likely to engage fellow racialized community members for emotional support (Blumberg *et al.*, 2015; Boukpessi *et al.*, 2021; Chiu *et al.*, 2018; Richman *et al.*, 2007). Unfortunately, many racialized GBM experience a disavowal of their sexual identity from members of their racialized communities (Sadika *et al.*, 2020), which limits access to stress-moderating emotional support (Bowleg, 2013; Kim and Allen, 2023; Nakamura *et al.*, 2013). Thus, racialized GBM may have increased mental health care needs (Choi *et al.*, 2013; English *et al.*, 2018; Jackson *et al.*, 2020; Layland *et al.*, 2022). However, it is unclear whether MHS use patterns in racialized GBM align more with white GBM or with trends observed in racialized communities.

In Canada, GBM represent one-half of people living with HIV (Government of Canada, 2022). HIV stigma represents an additional stressor for GBM living with HIV (GBMHIV) (Rzeszutek *et al.*, 2021; Smit *et al.*, 2012) which can also contribute to poor mental health. For example, a study of 671 Canadian GBMHIV found HIV stigma to be associated with higher suicidality (Ferlatte *et al.*, 2017). For racialized GBMHIV, homonegativity, including culturally rooted heteronormative norms; racism; and HIV stigma may all coexist and intersect in different configurations in the social milieus they navigate (Arnold *et al.*, 2014; Arscott *et al.*, 2020; Bogart *et al.*, 2011; MacCarthy *et al.*, 2021).

Intersectionality is a critical theoretical framework developed by Black feminists (Crenshaw, 1989; Hill Collins, 2019). It focuses on neglected groups whose experiences are often essentialized to those of a larger dominant group (Bowleg, 2012). Intersectionality examines how the interrelatedness and mutual construction of multiple systems of inequity (e.g., homonegativity, racism, HIV stigma) shape the experiences of people living at the intersections of such systems (Bowleg, 2013; Hill Collins, 2019). As an analytical strategy, it diverges from methods that treat social identities of individuals (e.g., sexual identity, racial identity, living with HIV) as additive (i.e., mutually exclusive), ahistorical and socially invariant (Bowleg, 2013; Cho *et al.*, 2013; Hancock, 2007; Nayak, 2021). It also refrains from using identities as determinants of outcomes. Instead, intersectionality emphasizes the role of power or its surrogates (e.g., discrimination) to dynamically accord identities certain social positions, consequently affecting outcomes (Hill Collins, 2019; Sievwright *et al.*, 2022).

Many studies on Black GBM in the US have leveraged intersectionality to better understand their disengagement from HIV care (Arnold *et al.*, 2014; Arscott *et al.*, 2020; Lutete *et al.*, 2022). However, few studies have used an intersectionality-based framework to evaluate MHS use in GBM. MHS represent avenues through which discrimination-related stress and its negative effects on mental health (Moody *et al.*, 2023) and on HIV care of GBM could be mitigated.

In this manuscript, our objectives are (1) to compare MHS use in different GBM groups (based on racial identity and HIV status) and (2) to estimate the mediating effect of self-reported interpersonal perceived discrimination (PD) between GBM groups and MHS use, using a quantitative intersectionality-based approach. Different quantitative methods have been used in intersectionality research (Bauer *et al.*, 2021). We utilize a modification of causal mediation analysis, the three-way decomposition of causal effects (VanderWeele, 2013), as proposed by Bauer and Scheim (2019), to meet our objectives.

## Methods

### Study population

We used baseline data (February 2017 to August 2019) from Engage, a prospective cohort study of GBM in Montreal, Toronto and Vancouver. GBM (cisgender and transgender) ≥16 years who reported sexual activity with another man in the previous 6 months and provided informed consent in French or English were enrolled. Participants were recruited by respondent-driven sampling (RDS), an adapted form of the chain referral method, in which peer referrals and self-reported network sizes of participants are recorded (Gile *et al.*, 2018). Engage study details have been described in detail elsewhere (Cox *et al.*, 2021; Hart *et al.*, 2021; Moore *et al.*, 2021).

At study visit, all participants completed computer-assisted self-administered questionnaires and underwent tests for HIV and other sexually transmitted and blood borne infections.

### Study definitions

**Exposure** was categorized into four groups. **Group 1:** white HIV-negative GBM, **Group 2:** white GBMHIV, **Group 3:** racialized HIV-negative GBM and **Group 4:** racialized GBMHIV. We operationalize the definition of racialization as used by the Government of Canada (2023a). Racialized GBM included Indigenous men, those listed as visible minorities under Canada’s Employment Equity Act (South Asian, Chinese, Korean, Japanese, Black, Filipino, Arab, Latin American, Southeast Asian and West Asian; Government of Canada, 1995) and those self-identifying as persons of colour.

Our categorization recognizes that homonegativity, racialization and HIV stigma are social phenomena which differentially affect the social positions of members of the exposure groups. Further, it emphasizes that the experiences of racialized GBMHIV cannot be understood as the sum of experiences of racialized HIV-negative GBM and white GBMHIV.

The **mediator** was PD scores measured as continuous values on a short version of the Everyday Discrimination Scale (EDS; Williams *et al.*, 1997), previously used in a national Canadian survey (Hyman *et al.*, 2019). In the first half of the EDS, PD is measured using five questions not bound to a timeframe and without attribution to sexual identity, racial identity or/and living with HIV. Questions range from an assessment of minor daily hassles (i.e., being treated with less respect or courtesy) to more consequential experiences (i.e., being threatened or harassed). Responses are scored on a 5-point Likert scale (1 – never, 5 – at least once a week). Total scores (range: 5–25) are calculated by summing responses over the five questions. Scores are directly proportional to the chronicity of PD (Lewis *et al.*, 2015). Since the score is attribution-free, we posited that the total score encapsulates the interconnectedness of discriminatory experiences. We used the total score for quantitative analyses. In the second half of the EDS, participants indicate all identities to which they attribute discrimination. We counted attributions to sexual identity, racial identity and HIV for each exposure group and examined these descriptively but not in quantitative analyses.

The **outcome** was MHS use in the prior 6 months, determined as a binary variable (yes/no). MHS use was defined as having seen or talked to an MHS provider (physician, psychologist, psychiatrist, social worker and community-based counsellor) about one’s mental or emotional health.

### Statistical analyses

We excluded GBM with missing data for the outcome (*n* = 63) or those diagnosed with HIV in the enrolment year or for the first time through the study (*n* = 15). The latter were excluded assuming their experiences of HIV stigma would be dissimilar to GBM who had lived longer with HIV.

Study population characteristics across the three cities were described with and without RDS adjustment. This adjustment accounts for participants’ self-reported peer network sizes and is city specific. It uses inverse probability weights to account for the likelihood of being sampled (i.e., to account for the higher probability of those with large network sizes being sampled, therefore, preventing selection bias from unduly affecting estimates) (McCreesh *et al.*, 2012). RDS-unadjusted estimates are presented whenever data combined across the three cities (i.e., three-city data) are used.

For the causal mediation analysis, three-city data were used. Conventionally, the experiences of white HIV-negative GBM (Group 1) are generalized to most GBM, particularly in research unrelated to HIV (Bowleg, 2013; Kulick, 2013; Sadika *et al.*, 2020). Here, Group 1 serves as the reference to facilitate the evaluation of results from the vantage point of underrepresented and differentially disprivileged GBM groups.

To fit a causal mediation model on cross-sectional baseline data, we made two assumptions regarding the temporality of measured variables. First, we assumed that sexual identity, racial identity and HIV status preceded PD (mediator). Due to being in an inequitable society, homonegativity, racism and HIV stigma are consequences of GBM identity, racialization and living with HIV. Thus, we believe our first assumption is valid. Second, we assumed that for most participants, PD (not time-bound) preceded the outcome and remained relatively unchanged during the assessment period of the outcome (prior 6 months). To our knowledge, there were no sociopolitical events in Montreal, Toronto and Vancouver 6 months prior to enrolment (i.e., between 2016 and 2019) that may have caused a significant change in average PD. Therefore, we believe that our second assumption is reasonable.

Traditional causal mediation analysis decomposes the total effect (TE) of the exposure on the outcome into a pure direct effect (PDE, unmediated effect) and a total indirect effect (TIE, mediated effect) (VanderWeele, 2013). This assumes that the mediator has the same effect on the outcome for all exposure groups. Discrimination is not experienced independent of historical and social contexts. Hence, PD may have different meanings and effects for exposure groups. The three-way decomposition further separates the TIE into a pure indirect effect (PIE) and a mediated interaction effect (MIE). Statistically, this introduces an interaction between the exposure and the mediator. This allows PD to have heterogeneous effects on the outcome for different exposure groups (Bauer and Scheim, 2019). Interpretations for PDE, PIE, MIE and TE are presented in Table 1.

**Table 1.** Interpretations of three-way decomposition of mediated effect estimates with a binary outcome

Effect	Interpretation	Application to given analysis^a^
**Pure direct effect (PDE)**	Direct unmediated effect. The odds ratio of the outcome for comparator versus reference exposure group, if both groups experienced the reference exposure group’s level of the mediator. The expected residual odds ratio of the outcome, if the comparator exposure group’s mediator level was set to the same level as that of the reference exposure group.	What would be the odds ratio of mental health services (MHS) use in racialized HIV-negative GBM, if the level of perceived discrimination (PD) in racialized HIV-negative GBM was set to the same lower level as that of white HIV-negative GBM?
**Pure indirect effect (PIE)**	Effect due to mediation only. The odds ratio of the outcome for comparator versus reference exposure group, if the level of the mediator in the comparator exposure group is as it would naturally occur (i.e., not fixed to an external investigator-defined value), compared to the mediator levels of the reference exposure group. Effect due to differential levels of the mediator.	What would be the odds ratio of MHS use in racialized HIV-negative GBM due to increased PD levels relative to white HIV-negative GBM, if the effect of PD on MHS use was of the same magnitude and direction as for white HIV-negative GBM?
**Mediated interaction effect (MIE)**	Effect due to mediation and interaction. The odds ratio of the outcome for the comparator versus reference exposure group explained by the mediator having different effects for the comparator exposure and reference exposure groups. Effect due to differential effect of the mediator.	What would be the additional odds ratio of PD on MHS use for racialized HIV-negative GBM compared to white HIV-negative GBM?
**Total effect (TE)**	Overall effect. Derived by multiplying together PDE, PIE and MIE. The odds ratio of the outcome in the comparator versus reference exposure group.	What is the odds ratio of MHS use in racialized HIV-negative GBM compared with white HIV-negative GBM?

*Source:* Adapted from Bauer and Scheim (2019).

a Application examples are provided using white HIV-negative GBM (Group 1) as the reference exposure group and racialized HIV-negative GBM (Group 3) as the comparator exposure group. Interpretations for white GBM living with HIV (white GBMHIV, Group 2) and racialized GBMHIV (Group 4) can be made similarly.

We used the imputation-based approach of natural effect models to operationalize the three-way decomposition (Vansteelandt *et al.*, 2012). This method estimates PDE and PIE directly without resorting to complex formulae, used in traditional three-way decomposition. EDS scores were standardized by mean-centring and dividing by the standard error of the scores’ distribution. We obtained odds ratios (ORs) for PDE, PIE, MIE and TE, comparing different exposure groups to Group 1, after controlling for confounders. Confounders were age, having a lifetime chronic mental health condition diagnosed >6 months ago (yes/no), Canadian citizenship (yes/no), being cisgender (yes/no) and city of enrolment. Conventionally, confounders are chosen to meet the assumptions required for causal mediation analysis, namely, control for exposure–outcome, exposure–mediator and mediator–outcome confounding, and no exposure-induced mediator–outcome confounding (no confounder for the mediator–outcome relationship is itself affected by the exposure) (VanderWeele, 2016). However, as noted in Table 1, potential outcomes are defined based on intervening on the mediator and not on the exposure (i.e., we consider racial identity and HIV seropositivity as non-modifiable). Thus, our interpretations in Table 1 hold under the assumptions of sufficient confounder control for the mediator–outcome relationship (VanderWeele and Robinson, 2014) and no exposure-induced mediator–outcome confounding. Primary findings are presented without including RDS weights, as no consensus exists on their incorporation in natural effect models (Avery *et al.*, 2021; Gile *et al.*, 2018), although there exists ongoing discussion (Yauck *et al.*, 2021).

We performed three sensitivity analyses to verify the robustness of our natural effect model findings. (1) We compared our primary results with those after accounting for RDS weights. (2) We considered socioeconomic factors (income, education and employment), social support and resilience as confounders of the mediator–outcome relationship. These could potentially be affected by the exposure and violate a key assumption of causal mediation analysis (i.e., no exposure-induced confounding). Therefore, they were not included in the primary model. Findings from models in which they were included as separate confounders (in addition to primary model confounders) were nonetheless compared to primary results. Mean scores on the Medical Outcomes Study Social Support Survey Instrument (Sherbourne and Stewart, 1991) and the Connor Davidson Resilience Scale-2 (Vaishnavi *et al.*, 2007) were used as measures of social support and resilience. (3) Finally, we compared primary results with those in which we lagged the outcome by 1 year. This was done to assess if a potential overlap between the assessment timeframes of the mediator and outcome (i.e., simultaneous or reversed temporality) biased the primary results. We did not treat this as the primary model for several reasons. There was a 29% loss to follow-up (LFU) between enrolment and first follow-up visit at 1 year, leading to an important loss of sample size and hence power. Moreover, the long lag between variables may dilute the estimated mediated effect. For this sensitivity analysis, we additionally accounted for LFU by using inverse probability weights (Willems *et al.*, 2018). This weighting may reduce bias due to selective LFU but also increases standard errors relative to an unweighted analysis, further decreasing power.

All analyses were performed in Stata 17.0 and R statistical software using the *RDS* and *medflex* packages. More details of the statistical procedures are provided in the supplementary section.

## Results

### Study population

We included 1127, 504 and 740 GBM participants from Montreal, Toronto and Vancouver, respectively. RDS-adjusted mean age of participants ranged between 34.8 years (95% CI: 32.6, 36.9; Toronto) and 36.9 years (95% CI: 35.5, 38.2; Montreal). Racialized GBM constituted 27.8% (95% CI: 22.6%, 33.1%; Montreal); 37.6% (95% CI: 30.5%, 44.6%; Toronto) and 45.6% (95% CI: 39.2%, 52.0%; Vancouver) of the study population. The proportions of GBMHIV were 13.8% (95% CI: 10.2%, 17.4%; Montreal), 19.1% (95% CI: 14.8%, 23.5%; Toronto) and 19.4% (95% CI: 13.8%, 24.9%; Vancouver). Mean duration of living with HIV was 17.5 years (95% CI: 14.7, 20.4; Montreal), 17.1 years (95% CI: 13.8, 20.3; Toronto) and 15.1 (95% CI: 12.4, 17.8; Vancouver). Approximately, one quarter of participants used MHS in the prior 6 months across all three cities (Montreal, 26.2%, 95% CI: 21.8%, 30.6%; Toronto, 30.1%, 95% CI: 24.1%, 37.2%; Vancouver, 24.7%, 95% CI: 19.6%, 29.8%).

Montreal had the highest proportion of white HIV-negative GBM (61.2%, 95% CI: 55.7%, 66.6%) and Toronto the highest proportion of white GBMHIV (14.5%, 95% CI: 10.8%, 18.2%). Vancouver had the highest proportion of both racialized HIV-negative GBM (38.1%, 95% CI: 31.9%, 44.2%) and racialized GBMHIV (7.5%, 95% CI: 3.7%, 11.4%) (Table 2).

**Table 2.** Characteristics of participants recruited across Montreal, Toronto and Vancouver in the Engage cohort study via respondent-driven sampling

	Montreal ( *n* = 1127)		Toronto ( *n* = 504)		Vancouver ( *n* = 740)	
	Unadjusted *n* (%)	RDS adjusted % 95% CI	Unadjusted *n* (%)	RDS adjusted % 95% CI	Unadjusted *n* (%)	RDS adjusted % 95% CI
**Mean age in years (SD)**	38.4 (13.6)	36.9 (35.5, 38.2)	33.8 (10.6)	34.8 (32.6, 36.9)	36.1 (12.6)	35.4 (33.6, 37.2)
**Sexual identity**						
Gay	925 (82.1)	75.3 (70.7, 79.9)	393 (77.9)	74.9 (68.5, 81.3)	633 (85.4)	80.7 (74.7, 86.6)
Bisexual	87 (7.7)	12.1 (8.6, 15.6)	21 (4.2)	10.8 (5.8, 15.8)	39 (5.3)	10.6 (5.8, 15.3)
Queer	62 (5.5)	5.0 (2.6, 7.5)	73 (14.5)	9.2 (4.8, 13.6)	44 (5.9)	3.7 (1.0, 6.6)
Pansexual	27 (2.4)	3.8 (2.0, 5.6)	13 (2.6)	3.0 (1.4, 4.7)	9 (1.2)	1.0 (0, 2.3)
Other^a^	26 (2.3)	3.7 (0.6, 6.9)	4 (0.8)	2.0 (0.0, 3.4)	15 (2.0)	4.0 (0.8, 7.2)
**Identify as cisgender**						
Yes	1042 (92.5)	88.7 (84.8, 92.5)	473 (93.8)	91.6 (88.8, 94.5)	707 (95.5)	93.4 (89.6, 97.2)
No	85 (7.5)	11.3 (7.4, 15.1)	31 (6.2)	8.4 (5.5, 11.2)	33 (4.5)	6.6 (2.8, 10.4)
**Education**						
≤High school	218 (19.3)	22.4 (18.0, 26.8)	51 (10.1)	19.4 (13.2, 25.8)	104 (14.1)	20.0 (14.2, 25.9)
>High school & <Bachelor’s degree	398 (35.3)	35.5 (30.4, 40.7)	159 (31.6)	35.9 (28.5, 43.3)	252 (34.1)	30.3 (24.2, 36.4)
≥Bachelor’s degree	511 (45.3)	42.1 (36.5, 47.6)	294 (58.3)	44.6 (36.9, 52.3)	384 (51.8)	49.7 (42.8, 56.5)
**Annual income (Canadian dollars)**						
<30,000	636 (56.4)	65.3 (60.4, 70.1)	236 (46.8)	55.5 (48.4, 62.7)	336 (45.4)	60.8 (54.6, 67.1)
30,000–50,000	277 (24.6)	20.4 (16.3, 24.4)	118 (23.4)	28.6 (22.4, 34.7)	155 (20.9)	18.3 (13.3, 23.3)
≥50,000	214 (19.0)	14.4 (11.0, 17.8)	150 (29.8)	15.9 (10.5, 21.2)	249 (33.7)	20.9 (15.8, 26.0)
**Employed**						
Yes	773 (68.6)	61.7 (56.5, 66.9)	368 (73.0)	54.1 (46.9, 61.3)	569 (76.9)	70.8 (64.6, 77.0)
No	354 (31.4)	38.3 (33.1, 43.5)	136 (27.0)	45.9 (38.7, 53.1)	171 (23.1)	29.2 (23.0, 35.4)
**Student**						
Yes	310 (27.5)	29.8 (24.8, 34.8)	112 (22.2)	27.9 (21.1, 34.8)	144 (19.5)	26.7 (21.2, 32.2)
No	817 (72.5)	70.2 (65.2, 75.2)	392 (77.8)	72.0 (65.2, 78.9)	596 (80.5)	73.3 (67.8, 78.8)
**Canadian citizen**						
Yes	919 (81.5)	74.9 (70.0, 79.9)	399 (79.2)	71.2 (64.1, 78.3)	583 (78.8)	68.6 (62.5, 74.7)
No	208 (18.5)	25.1 (20.1, 30.0)	105 (20.8)	28.8 (21.7, 35.9)	157 (21.2)	31.4 (25.3, 37.5)
**Racialized^b^**						
Yes	243 (21.6)	27.8 (22.6, 33.1)	173 (34.3)	37.6 (30.5, 44.6)	252 (34.0)	45.6 (39.2, 52.0)
No	884 (78.4)	72.2 (66.9, 77.4)	331 (65.7)	62.4 (55.4, 69.5)	488 (66.0)	54.4 (48.0, 60.8)
**Living with HIV**						
Yes	198 (17.6)	13.8 (10.2, 17.4)	95 (18.8)	19.1 (14.8, 23.5)	125 (16.9)	19.4 (13.8, 24.9)
No	929 (82.4)	86.2 (82.6, 89.8)	409 (81.2)	80.9 (76.5, 85.2)	615 (83.1)	80.6 (75.1, 86.2)
**Intersectional groups**						
White HIV-negative (Group 1)	718 (63.7)	61.2 (55.7, 66.6)	260 (51.6)	47.9 (40.7, 55.2)	398 (53.8)	42.6 (36.2, 48.9)
White living with HIV (Group2)	166 (14.7)	11.0 (7.7, 14.3)	71 (14.1)	14.5 (10.8, 18.2)	90 (12.2)	11.8 (7.3, 16.3)
Racialized HIV-negative (Group 3)	211 (18.7)	25.0 (19.8, 30.3)	149 (29.6)	32.9 (26.0, 39.9)	217 (29.3)	38.1 (31.9, 44.2)
Racialized living with HIV (Group 4)	32 (2.8)	2.8 (1.3, 4.3)	24 (4.8)	4.6 (2.2, 7.1)	35 (4.7)	7.5 (3.7, 11.4)
**Has a primary healthcare provider**						
Yes	765 (67.9)	60.0 (54.9, 65.1)	408 (80.9)	73.0 (65.2, 80.7)	524 (70.8)	66.4 (60.3, 72.4)
No	362 (32.1)	40.0 (54.9, 65.1)	96 (19.1)	27.0 (19.3, 34.8)	216 (29.2)	33.6 (27.5, 39.7)
**Has medical insurance**						
Yes	834 (74.0)	68.2 (63.3, 73.2)	328 (65.1)	61.8 (54.0, 69.6)	524 (70.8)	63.1 (56.8, 69.4)
No	293 (26.0)	31.8 (26.8, 36.7)	176 (34.9)	38.2 (30.4, 45.9)	216 (29.2)	36.9 (30.6, 43.2)
**Has a chronic mental health condition^c^ (for ≥6 months)**						
Yes	398 (35.3)	33.1 (28.1, 38.1)	186 (36.9)	39.8 (32.8, 46.9)	272 (36.8)	29.9 (24.0, 35.8)
No	701 (62.2)	66.9 (61.8, 71.9)	305 (60.5)	60.2 (53.1, 67.2)	456 (61.6)	70.1 (64.2, 76.0)
Missing	28 (2.5)	–	13 (2.6)	–	12 (1.6)	–
**Self-rated mental health (past 6 months)**						
Good to excellent	870 (77.2)	74.4 (69.3, 79.5)	335 (66.5)	68.2 (61.4, 75.1)	515 (69.6)	72.5 (66.7, 78.3)
Fair to poor	252 (22.4)	25.6 (20.5, 30.6)	168 (33.3)	31.8 (24.9, 38.6)	224 (30.3)	27.5 (21.7, 33.3)
Missing	5 (0.4)	–	1 (0.2)	–	1 (0.1)	–
**Ease to obtain mental health services**						
Easy or very easy	660 (58.6)	71.4 (65.7, 77.0)	272 (54.0)	75.9 (68.5, 83.4)	449 (60.7)	77.3 (71.6, 82.9)
Difficult or very difficult	101 (8.9)	10.9 (6.9, 14.8)	62 (12.3)	10.8 (5.9, 15.7)	56 (7.6)	10.1 (5.7, 14.5)
Don’t know	143 (12.7)	17.8 (12.9, 22.7)	54 (10.7)	13.2 (6.7, 19.8)	69 (9.3)	12.6 (8.5, 16.7)
Missing	223 (19.8)	–	116 (23.0)	–	166 (22.4)	–
**Mental health services use (past 6 months)**						
Yes	317 (28.1)	26.2 (21.8, 30.6)	180 (35.7)	30.1 (24.1, 37.2)	231 (31.2)	24.7 (19.6, 29.8)
No	810 (71.9)	73.8 (69.4, 78.2)	324 (64.9)	69.3 (62.8, 75.9)	509 (68.8)	75.3 (70.2, 80.4)

RDS: respondent-driven sampling; 95% CI: 95% confidence intervals; SD: standard deviation.

a Other (sexual identity) includes straight (*n* = 7), questioning (*n* = 7), asexual (*n* = 2), two-spirit (*n* = 15) and responses that do not include any of the categories listed (*n* = 15).

b Racialized includes Indigenous peoples, those listed as visible minorities under the Employment Equity Act of Canada or those that self-identify as persons of colour.

c Chronic mental health conditions include any of the following mental health provider diagnosed conditions: substance use disorder, anxiety disorder, post-traumatic stress disorder (PTSD), attention deficit disorder or attention deficit hyperactivity disorder (ADD or ADHD), bipolar disorder, borderline personality disorder or major depressive disorder.

### PD scores and MHS use in the prior 6 months by exposure groups

The EDS demonstrated good internal consistency (study Cronbach’s *α* > 0.90) for all three cities. Racialized GBMHIV in Montreal had the lowest mean EDS score (7.2, 95% CI: 5.8, 8.6), and racialized GBMHIV in Toronto had the highest (13.0, 95% CI: 9.2, 16.7).

Between cities, MHS use was variable across GBM groups. In Montreal, MHS use was highest in racialized GBMHIV (33.6%, 95% CI: 11.2%, 56.1%); in Toronto, it was highest in white GBMHIV (35.0%, 95% CI: 16.8%, 53.2%) and racialized HIV-negative GBM (35.3, 95% CI: 23.0%, 47.5%); and in Vancouver, it was highest in white GBMHIV (37.7%, 95% CI: 20.9%, 54.5%) (Table 3).

**Table 3.** Mental health services use in the past 6 months by GBM enrolled in the Engage cohort study by intersectional categories and mean perceived discrimination scores measured on the Everyday Discrimination Scale (EDS)

			Montreal			Toronto			Vancouver		
				MHS use P6M			MHS use P6M			MHS use P6M	
	Overall unadjusted mean EDS score^a^ (SD)	Overall unadjusted MHS use P6M^b^ *n* (%)	RDS adjusted mean EDS score (95% CI)	Unadjusted *n* (%)	RDS adjusted % (95% CI)	RDS adjusted mean EDS score (95% CI)	Unadjusted *n* (%)	RDS adjusted % (95% CI)	RDS adjusted mean EDS score (95% CI)	Unadjusted *n* (%)	RDS adjusted % (95% CI)
**Group 1**	8.4 (3.9)	411 (29.9)	7.5 (7.0, 8.0)	198 (27.6)	28.0 (22.3, 33.7)	9.1 (8.2, 10.0)	91 (35.0)	26.9 (18.0, 35.8)	8.9 (8.2, 9.7)	122 (30.6)	26.0 (18.2, 33.8)
**Group 2**	8.5 (4.4)	132 (40.4)	7.6 (6.7, 8.5)	55 (33.1)	28.7 (16.6, 40.8)	7.6 (5.7, 9.6)	33 (46.5)	35.0 (16.8, 53.2)	8.7 (7.3, 10.2)	44 (48.9)	37.7 (20.9, 54.5)
**Group 3**	10.3 (5.0)	155 (26.9)	9.6 (8.3, 10.9)	55 (26.1)	19.7 (11.7, 27.6)	10.3 (8.8, 11.9)	46 (30.8)	35.3 (23.0, 47.5)	9.6 (8.6, 10.7)	54 (24.9)	20.0 (12.9, 27.1)
**Group 4**	10.1 (4.8)	30 (32.9)	7.2 (5.8, 8.6)	9 (28.1)	33.6 (11.2, 56.1)	13.0 (9.2, 16.7)	10 (41.7)	22.9 (0, 49.8)	7.7 (5.6, 9.7)	11 (31.4)	20.4 (0, 43.8)

**Group 1**: white HIV-negative, **Group 2**: white living with HIV, **Group 3**: racialized HIV-negative, **Group 4**: racialized living with HIV.

MHS use P6M: mental health services use in the past 6 months; RDS: respondent-driven sampling; SD: standard deviation; 95% CI: 95% confidence interval.

Overall represents the total study population combining across the three cities, these are not RDS weighted as RDS weights are city specific.

a EDS range: 5–25.

b Total participants: **Group 1**: *n* = 1376, **Group 2**: *n* = 327, **Group 3:** *n* = 577, **Group 4**: *n* = 91.

There were 64 missing values for EDS scores (27 for Group 1; 8 for Group 2; 24 for Group 3; 5 for Group 4). If a participant indicated that they preferred not to respond to any of the five items on the EDS, the total score was not calculated, and that participant was assigned a missing value.

### Attributions of discrimination

We used three-city data. As mentioned, these data do not serve an analytical purpose but are presented to showcase the distribution of discriminatory experiences attributed to sexual identity, racial identity and HIV status.

In Group 1 (white HIV-negative GBM), 46.6% reported a single form of discrimination mostly attributed to sexual identity. In Group 2 (white GBMHIV), 20.5% reported two forms of discrimination mostly attributed to sexual identity and HIV status. In Group 3 (racialized HIV-negative GBM), 38.3% reported two forms of discrimination attributed mostly to sexual and racial identities, whereas in Group 4 (racialized GBMHIV), 17.6% reported discrimination attributed to sexual identity, racial identity and HIV status. However, across the four groups, attributions to discrimination based on sexual identity, racial identity and HIV status were varied. Individuals with multiply marginalized identities did not necessarily attribute discrimination to all aspects of their identities. Moreover, 25.3% (Group 4) to 47.1% (Group 1) of participants reported no experiences of discrimination based on their sexual identity, racial identity and HIV status (Table 4).

**Table 4.** Attribution(s) of discrimination to sexual identity, racial identity and HIV status across exposure groups using data combined across the three cities

	Group 1 (*n* = 1376) *n* (%)	Group 2 (*n* = 327) *n* (%)	Group 3 (*n* = 577) *n* (%)	Group 4 (*n* = 91) *n* (%)
**No discrimination^a^**	653 (47.5)	154 (47.1)	179 (31.0)	23 (25.3)
**One attribution**	641 (46.6)	94 (28.8)	169 (29.3)	28 (30.8)
Due to sexual identity only	629	60	70	14
Due to racial identity only	12^b^	5^b^	98	11
Due to HIV status only	0	29	1^c^	3
**Two attributions**	66 (4.8)	67 (20.5)	221 (38.3)	22 (24.2)
Due to sexual and racial identities	58^b^	1^b^	213	14
Due to sexual identity and HIV status	8^c^	66	8^c^	5
Due to racial identity and HIV status	0	0	0	3
**Three attributions**	2 (0.2)^b^^,^^c^	8 (2.4)^b^	3 (0.5)^c^	16 (17.6)
**Missing^d^**	14 (1.0)	4 (1.2)	5 (0.9)	2 (2.2)

Patterns for each city were consistent with those presented for the total study population here.

Note: HIV status indicates HIV seropositivity.

**Group 1**: white HIV-negative, **Group 2**: white living with HIV, **Group 3**: racialized HIV-negative, **Group 4**: racialized living with HIV.

a No discrimination includes participants who marked never to all five questions on the first part of the EDS (i.e., total EDS score of 5) or those that indicated not having faced discrimination due to their sexual identity, racial identity and HIV status in the second part of the EDS.

b Attribution to racial identity among groups that included white GBM (*n* = 86): 32 were not Canadian citizens; 29 identify their ethnicity as English Canadian, 8 French Canadian, 39 of Eastern or Western European ancestry and 10 as mixed ethnicity; 66 were primarily English speakers, 13 primarily French speakers and 7 spoke either Spanish, Russian, Slovak, Chechen, Turkish or Portuguese primarily. No information on Jewish/Islamic heritage or political affinity was available. None identified as persons of colour.

c Attribution to HIV status among groups that included HIV-negative GBM (*n* = 22): 5 were born in the 1960s, 14 were born between 1978 and 1987 and 3 in the 1990s; 12 were born either in Syria, Rwanda, Lebanon, Sri Lanka, Cameron, Slovakia, Turkey, Mexico, Philippines or Brazil.

d There is a discrepancy in the number of missing values with Table 3 (*n* = 64). This is because participants that did not respond to any question on the first part of the EDS were marked as a missing value when calculating the total EDS score but could still indicate whether they experienced discrimination due to their sexual identity, racial identity or HIV status in the second part of the EDS.

Note that enumerating multiple forms of discrimination is not equivalent to an intersectionality-based approach. Attributions have been presented to highlight that GBM who experience discrimination because of multiply marginalized identities do not experience an ambiguous form of ‘intersectional’ discrimination. GBM may be able to recall ‘identifiable’ forms of discrimination (i.e., homonegativity, racism and HIV stigma) due to existing systems of inequity to different extents (Lewis *et al.*, 2015). Thus, enumeration may help to identify actionable items for intervention. However, exclusive enumeration of multiple (but individual) forms of discrimination may lend itself to an understanding that frames experiences of discrimination as compartmentalized and therefore additive. An intersectionality-based approach acknowledges that experiences of discrimination due to different systems of inequity overlap with each other (i.e., cannot be examined independent of each other) to different degrees and are dynamically constructed within historical and social contexts.

### Natural effect model estimates

Group 2 (white GBMHIV) had higher overall MHS use odds (TE [OR] 1.71; 95% CI: 1.27, 2.29) compared with Group 1 (white HIV-negative GBM). PD did not significantly mediate MHS use (PIE [OR], 1.02; 95% CI: 0.98, 1.05). As evinced by the PDE, if PD levels were reduced to that of Group 1, higher MHS use odds in Group 2 (OR, 1.68; 95% CI: 1.27, 2.24) would still be observed.

Relative to Group 1, Group 3 (racialized HIV-negative GBM) did not have significantly different overall MHS use odds (TE [OR], 0.95; 95% CI: 0.74, 1.22). Higher PD levels (compared with Group 1) were associated with higher odds of MHS use (PIE [OR], 1.09; 95% CI: 1.02, 1.17). If PD levels were reduced to that of Group 1, we estimate the odds of MHS use in Group 3 would concomitantly reduce, albeit not statistically significantly (PDE [OR], 0.84; 95% CI: 0.65, 1.09).

Relative to Group 1, TE (OR), 1.23, 95% CI: 0.73, 2.01; PIE (OR), 1.08, 95% CI: 0.99, 1.17 and PDE (OR), 1.23, 95% CI: 0.73, 2.15 for Group 4 (racialized GBMHIV) were not significantly different.

PD did not appear to have heterogeneous effects on MHS use for Groups 2, 3 and 4, relative to Group 1. This is indicated by non-significant MIE estimates (Table 5).

**Table 5.** Odds ratios for MHS use in the past 6 months across different intersectional groups, with perceived discrimination measured on the Everyday Discrimination Scale as the mediator

	Reference: Group 1 Comparison: Group 2	Reference: Group 1 Comparison: Group 3	Reference: Group 1 Comparison: Group 4
**Total effect (TE)**	1.71 (1.27, 2.29)*	0.95 (0.74, 1.22)	1.23 (0.73, 2.01)
**Pure direct effect (PDE)**	1.68 (1.27, 2.24)*	0.84 (0.65, 1.09)	1.23 (0.73, 2.15)
**Pure indirect effect (PIE)**	1.02 (0.98, 1.05)	1.09 (1.02, 1.17)*	1.08 (0.99, 1.17)
**Mediated interaction effect (MIE)**	1.00 (0.98, 1.02)	1.04 (0.93, 1.15)	0.92 (0.76, 1.13)

* Indicates statistically significant results.

**Group 1**: white HIV-negative, **Group 2**: white living with HIV, **Group 3**: racialized HIV-negative, **Group 4**: racialized living with HIV.

**Confounders adjusted for in the model**: age, city, having a chronic mental health condition, Canadian citizenship and cisgender status.

Estimates when missing data (for EDS and having a chronic mental health condition) were multiply imputed using chained equations are comparable to those presented here.

### Sensitivity analysis findings

When we included RDS weights, there were no changes in the point estimates for TE, PDE, PIE or MIE. This may indicate that point estimates are not influenced by participants’ network sizes or may reflect the conditional nature of mediation models (in contrast to simple means and proportions, where RDS weighting is recommended). However, it may also indicate that the distribution of factors affecting PD and MHS use is homogenous across exposure groups. When additional confounders for socioeconomic indicators, social support or resilience were included separately, the difference in point estimates from Table 5 estimates varied between 0% and 5%. These factors, as measured in the Engage study, do not appear to be strong confounders. When the outcome was lagged by 1 year, PDE estimates (and consequently TE estimates) for Group 2 were reduced by 26%. We are unsure of the reasons for these reductions. However, these reductions must be interpreted cautiously; estimates could be biased if the underlying LFU mechanism depends on variables not available in the dataset such as incarceration (i.e., missingness is not at random) (Li *et al.*, 2013). The difference for other estimates varied between 0% and 9.7% across exposure groups. PIE remained the same for all groups, highlighting the consistency of PD’s effects on MHS use.

Results from sensitivity analyses are presented in Supplementary Tables S2–S6.

## Discussion

Using a quantitative intersectionality approach (Bauer and Scheim, 2019; Public Health Agency of Canada, 2022), we found MHS use to be higher among white GBMHIV but not significantly different for racialized GBM (irrespective of HIV status), compared with white HIV-negative GBM. Additionally, relative to white HIV-negative GBM, PD was a significant mediator of MHS use only for racialized HIV-negative GBM.

Higher MHS use among white GBMHIV compared with white HIV-negative GBM was not mediated by PD. This is explained by comparable PD levels between the two groups. In Canada, unemployment and lower income, both associated with increased stress (American Psychological Association, 2017; Baum *et al.*, 1986), are more prevalent among GBM than heterosexual men (Kinitz *et al.*, 2023; Waite *et al.*, 2019). Further, in the current sample, we observed lower employment and income among white GBMHIV relative to white HIV-negative GBM (Supplementary Table 7). Hence, higher MHS use in white GBMHIV could represent one means of coping with structural inequity-related stressors. Recent reviews call for equity-promoting labour interventions for GBM and other people living with HIV at structural levels (policy, community and institutional levels) (Kinitz *et al.*, 2023; Maulsby *et al.*, 2020). It may be worthwhile to consider how MHS may be integrated into such interventions. Additionally, the EDS only assesses interpersonal discrimination, and future studies should include structural discrimination measures, to better elucidate its effect on MHS use among different groups of GBM.

Our finding that increased PD is associated with higher MHS use for racialized HIV-negative GBM aligns with previous studies (Burgess *et al.*, 2007; Evans and Sheu, 2019; Richman *et al.*, 2007). However, these studies take a unidimensional approach considering only racial or sexual identity. We add to existing literature without assuming *a priori* that racialized HIV-negative GBM rank one identity over the other. Although not statistically significant, lower TE for racialized HIV-negative GBM (Group 3) compared with white HIV-negative GBM (Group 1) indicates that MHS use among racialized HIV-negative GBM may partly mirror underutilization observed in racialized communities (Chiu *et al.*, 2018; Richman *et al.*, 2007). Cultural norms influence MHS use patterns in racialized individuals in the general population (Boukpessi *et al.*, 2021; Chiu *et al.*, 2018). Thus, while higher MHS use among racialized HIV-negative GBM may represent one coping strategy for increased PD-related stress, the impact that cultural norms may have on this association also needs to be considered.

Racialized individuals (in the general population) have also been noted to use MHS primarily when self-management of mental/emotional health becomes untenable (Boukpessi *et al.*, 2021; Chiu *et al.*, 2018; Richman *et al.*, 2007). Although it is unclear if racialized GBM (irrespective of HIV status) use MHS as a last resort, addressing this research gap may be crucial to develop timelier MHS for multiply marginalized individuals. Another consideration is that racialized individuals experiencing chronic discrimination are more likely to have heightened vigilance for discrimination (Brondolo *et al.*, 2018). In such individuals, ambiguous experiences may then be perceived as discriminatory (Lewis *et al.*, 2015), cyclically exacerbating stress. Assuming the interrelatedness of discriminatory experiences (i.e., experiencing discrimination as a continuum rather than as discrete unrelated events because of the non-divisibility of identities in multiply marginalized GBM) increases their chronicity, it is possible that racialized GBM have increased vigilance for discrimination. Hence, for mental health practitioners working with racialized GBM, the extent to which both culture and increased vigilance affect MHS use may be important considerations.

The paucity and limited funding of GBM-affirmative MHS and predominance of medical models in mental healthcare (that disregards social context) are systemic barriers previously identified to deoptimize MHS for GBM (McIntyre *et al.*, 2011). Recently, some improvements on these barriers have been made. In 2022, the Canadian federal government announced a $100 million plan for Two-Spirit, Lesbian, Gay, Bisexual, Transgender, Queer/Questioning, Intersex, Asexual+ communities (The Globe and Mail, 2022), and intersectionality-guided psychotherapeutic approaches are emerging (Adames *et al.*, 2018; Jackson *et al.*, 2020; Nayak, 2021). However, the impact these measures will have on discrimination-related stress among GBM, especially those with multiple marginalized identities, remains to be seen. Individual-level intersectionality-based interventions without macrolevel reforms are unlikely to sustainably mitigate discrimination-related stress in GBM. Concurrently, interventions such as those that address homonegativity in racialized communities, and racism and HIV stigmatization in GBM communities are required (Sievwright *et al.*, 2022). An often-overlooked aspect of intersectionality is its emphasis on building coalitions across marginalized groups to collectively oppose overlapping systems of inequity (Carastathis, 2013; Sievwright *et al.*, 2022). Going forward, it may be useful for researchers to develop and evaluate coalitional anti-discriminatory multilevel interventions.

Our findings have several limitations that merit discussion. Our analyses were contingent on data available in the Engage Cohort Study (CIHR Canadian HIV Trials Network), whose primary aims were different from the objectives addressed in this manuscript. To improve statistical power, we grouped multiple ethnicities of non-European descent as racialized. Despite this, we were inadequately powered to make definitive inferences about Group 4 and about the MIEs for any group. While racialized GBM across ethnicities may share some cultural commonalities and similar discriminatory experiences (Giwa and Han, 2022; Sadika *et al.*, 2020), we are cognizant that grouping them risks homogenizing them. We advocate for larger studies with oversampling of GBM with non-European ethnicities. As the EDS was administered only at baseline, we cannot be definitive regarding the temporality in our primary analysis. However, we found consistent results when the outcome was lagged by 1 year, making it less likely that the primary findings were affected by reverse causation. Longitudinal PD measurements may further enrich our understanding of its time-varying effects. The EDS was not developed for intersectional analysis, and certain items on the scale correspond more to specific racialized experiences than those related to homonegativity or HIV stigmatization. Thus, the EDS may not be suited for comparison across multiple social categories (Harnois *et al.*, 2019). Additionally, the overall EDS score may encapsulate experiences to other forms of discrimination (e.g., ableism). We recommend the development of better quantitative tools to measure intersecting forms of discrimination, while cautioning against positivist stances that assume intersectional experiences represent a single fixed reality (Bowleg, 2008). Although the MIE allows for PD to vary heterogeneously between exposure groups, it estimates a constant within-group average effect of PD. However, even within the same group, PD may be shaped by the degree of identification with one’s social identities and their importance to self-image (Richman *et al.*, 2007), and as observed in Table 4, within-group experiences of PD are varied. Mixed methods studies that can generate both between-group and within-group average effects while also detailing the complexity and diversity of within-group PD experiences may be more suited for intersectionality-based research (Hankivsky and Grace, 2015). We note that our primary analysis relied on estimates that were not RDS-adjusted, which in the strictest view, limits our findings to the study sample. However, as our sensitivity analysis shows, adjusted estimates were comparable to unadjusted estimates indicating that our findings may be generalizable to the larger population of GBM living in Montreal, Toronto or Vancouver. Given that best statistical practices for inclusion of RDS weights in the specific context of natural effect models have not been established, the concordance between RDS unadjusted and adjusted estimates is reassuring. Our results show a positive association between PD and MHS use in racialized HIV-negative GBM. However, previous research has also indicated that discrimination may impair the accomplishment of cognitively demanding tasks (Richman and Lattanner, 2014) like MHS use. Therefore, it is possible that discrimination in different contexts may have different associations with MHS use.

The present study found that discrimination may be associated with MHS use at both baseline and 1-year follow-up. Longer longitudinal studies investigating the effects of intersecting forms of discrimination on MHS use trends among GBM are warranted. Simultaneously, we present natural effect models as a viable method for three-way decomposition, to reveal underlying mechanisms not apparent through the sole examination of TEs. However, more research to improve quantitative intersectionality measures and methodology is required.

## Supporting information

Marbaniang et al. supplementary material Marbaniang et al. supplementary material

## Data Availability

The datasets generated and/or analysed during the current study are not publicly available due to privacy concerns for this ongoing cohort study. Deidentified participant data used in this analysis are stored at the British Columbia Centre for Excellence in HIV/AIDS (BCCFE). For information regarding these databases, please contact Joseph Cox, a principal investigator on the Engage Study.

## Supplementary material

The supplementary material for this article can be found at https://doi.org/10.1017/S2045796024000143.
