# Supplementary material for: Using an intersectionality-based approach to evaluate mental health services use among gay, bisexual and other men who have sex with men in Montreal, Toronto and Vancouver
Source: Epidemiol Psychiatr Sci. 2024 Mar 5;33:e10. doi: 10.1017/S2045796024000143 (PMC10940056; doi:10.1017/S2045796024000143)
Supplement: Marbaniang et al. supplementary material [file S2045796024000143sup001.docx]

**Supplementary files**

**Glossary of terms**

1. **Homonegativity:** Having disdain, negative thoughts or attitudes, and discomfort around gay, bisexual, or lesbian individuals. It can broadly be classified into moralistic homonegativity, which is adverse attitudes towards homosexuality, and intolerance which is personal rejection of homosexuals as a group based on out-group prejudice (Doebler, 2015).
2. **Coping:** Process-oriented efforts to regulate emotion, cognition, behaviour, physiology, and the environment in response to events or circumstances appraised as stressful. Coping is context specific and distinct from more stable dispositions that can serve as coping resources (e.g., high self-esteem, optimism) (Lazarus and Folkman, 1984; Major et al., 2017)
3. **Racialization:** Racialization is the complex and contradictory process through which groups come to be designated as being part of a particular "race" and on that basis subjected to differential and/or unequal treatment. While white people are also racialized, this process is often rendered invisible or normative to those designated as white. As a result, white people may not see themselves as part of a race but still maintain the authority to name and racialize "others.” Historically, it has been white people who hold the social, political, and economic power to name and categorize people of colour and Indigenous peoples due to colonial history; in many countries, whiteness is maintained as the “norm” that other races are measured against. White-skinned people doing the naming/categorizing often categorize themselves as white or Caucasian or they may think of themselves as "raceless" and "normal." This "normalcy" is defined by the assumed "otherness" or "abnormality" of people of colour (Alberta Civil Liberties Research Centre, 2021).
4. **Racism:** Racism is a form of prejudice that assumes that the members of racial categories have distinctive characteristics and that these differences result in some racial groups being inferior to others. Racism generally includes negative emotional reactions to members of the group, acceptance of negative stereotypes, and racial discrimination against individuals; in some cases, it leads to violence (American Psychological Association, 2023). Racism also includes institutional practices which treats people differently because of their colour or ethnicity (Government of Canada, 2023a).
5. **HIV stigma**: HIV stigma is negative attitudes and beliefs about people living with HIV, which may result in HIV discrimination i.e., treating people living with HIV less favorably than those without HIV (Centers for Disease Control and Prevention, 2021) . Discrimination can include behaviours where they affect the enjoyment of rights for people living with HIV, as well as physical abuse, denial of health or social services, denial or loss of employment or education opportunities or even arrest. It can also be enshrined in criminal laws, travel restrictions, mandatory testing, and employment restrictions (UNAIDS, 2022).
6. **Stigmatized group:** A group of individuals that possess a common attribute culturally understood as devalued and discrediting – applied by another more powerful group of individuals. Rooted in normative judgments, stigmatized individuals are reduced from a complex whole and “usual” person to the single tainted and discounted attribute/trait. Individuals belonging to stigmatized groups are subject to a combination of negative stereotyped beliefs, prejudiced attitudes, and discriminatory behavior, by other non-stigmatized individuals and institutions (Fudge Schormans, 2014)

| **Supplementary Figure 1: A simplified scheme to illustrate different forms of discrimination and their intersections that GBM may experience for our analyses** | | |
| --- | --- | --- |
|  | **No HIV stigmatization** | **HIV stigmatization from a majority HIV-negative heterosexual society & HIV-negative GBM** |
| **No Racism** | White GBM  HIV-negative | White GBM living with HIV |
| **Racism from a majority heterosexual White society & White GBM** | Racialized GBM HIV-negative | Racialized GBM living with HIV |
| GBM may experience different forms of discrimination  depending on their identity(ies). Importantly, oppression and privilege may be experienced simultaneously. For example, a racialized GBM may experience oppressing systems of racism and homophobia but may have privileges of HIV-negative GBM. Whereas a White GBM living with HIV is privileged by not being racialized but may be oppressed systematically by HIV-stigmatization. | | |

Homonegativity from majority White heterosexual society & one’s predominantly heterosexual racialized community

Homonegativity from majority heterosexual society

**Brief overview of natural effect models for causal mediation analysis**

**
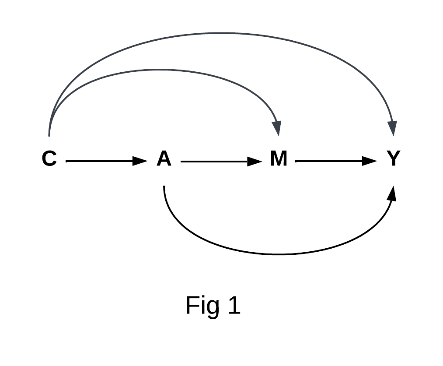
Causal mediation analysis**

Let *A* in Fig 1 represent the exposure of interest, $M$the mediator, $Y$the outcome and $C$ a set of confounders. We assume there is no unmeasured confounding (i.e., set $C$ includes all confounders for the exposure-mediator, mediator-outcome, and exposure-outcome relationships). Additionally, we assume that there are no variables $L$ in set $C$that are effects of $A$ and confounders of the mediator-outcome relationship (VanderWeele, 2016).

Causal mediation utilizes a potential outcomes or counterfactual framework. For each subject, we define $Y_{a,m}$ as the counterfactual outcome we would have observed, if counter to fact, the exposure $A$ were set to 𝑎 and mediator $M$ set to $m$. Similarly, $M_{a}$ represents the counterfactual value of the mediator, if counter to fact, $A$ were set to $a$ (Lange et al., 2012). Direct (unmediated) and indirect (mediated) effects can then be described in terms of *nested counterfactuals.* Thus, $Y_{a*, M_{a}}$denotes the outcome that would be observed if $A$ were set to $a^{*}$and $M$ were set to the value it would have taken if $A$ were set to $a.$ To estimate the natural direct effect (changing $a$ to $a^{*}$*)*, we compare $Y_{a, M_{a}}$ and $Y_{a*, M_{a}}$. Similarly, to estimate the natural indirect effect, we compare $Y_{a*, M_{a}}$ and $Y_{a*, M_{a*}}$. The word “natural” refers to the fact that we let the mediator take the value it would take naturally when the exposure is set to $a$. Natural direct and indirect effects can be estimated conditionally (i.e., within levels of covariates) or marginally. They can be expressed as mean difference or ratios (risk ratio, odds ratio) (Lange et al., 2012; Vansteelandt et al., 2012).

Total effect can be represented as the sum (for linear models) or product (for risk and odds ratios) of the natural direct and indirect effects. The natural direct effect is also called the pure direct effect and the natural indirect effect is also called the total indirect effect (VanderWeele, 2013).

**Traditional approach for causal mediation analysis**

Conventionally, the mediation formula is used to estimate natural direct and indirect effects, by first specifying separate models for the outcome and the mediator. The formula corresponds to a form of standardization, estimating the mean value of the outcome in each stratum defined by the mediator and confounders among the individuals with treatment $a^{*}$ but weighting these by the likelihood of each mediator value among individuals with treatment 𝑎 (Lange et al., 2012; Loeys et al., 2013; Vansteelandt et al., 2012).

For instance, $E\left[ Y_{a^{*}, M_{a}} | C=c \right]$ is estimated using Equation 1.

$\sum_{m} E\left[ Y | A=a^{*}, M=m, C=c \right]P(M=m|A=a, C=c)$ ……………………………………………Equation 1

When the outcome and the mediator follow linear models, i.e., $E\left[ Y | A=a, M=m, C=c \right]= \alpha_{0}+ \alpha_{1}a+ \alpha_{2}m+ \alpha_{3}c$ and $E\left[ M | A=a, C=c \right]=\beta_{0}+\beta_{1}a+\beta_{2}c$ then equations for natural direct, i.e., $E\left[ {(Y}_{a, M_{a}}-Y_{a*, M_{a}} \right)\left| C \right]$ and indirect effects, i.e., $E[(Y_{a^{*}, M_{a}}- Y_{a^{*},M_{a^{*}}})|C$] can be simplified to $\alpha_{1}(a-a^{*})$ and $\alpha_{2}\beta_{1}\left( a-a^{*} \right)$, respectively (Lange et al., 2012).

However, when the outcome or mediator are less suited to be modeled using a linear model (e.g., binary outcomes or non-normally distributed mediator), unless additional simplifying assumptions are made, the resulting expressions for natural direct and indirect effects depend on values of exposures $a$*a*nd $a^{*}$ and confounders in a complicated way (Lange et al., 2012; Tchetgen Tchetgen, 2014; VanderWeele, 2016; Vansteelandt et al., 2012). This makes results challenging to report as separate findings at each exposure or confounder value would require reporting (Vansteelandt et al., 2012).

**Natural effect models**

Natural effect models simultaneously estimate natural direct and indirect effects in the same model, without depending on complex formulae of the exposure or confounder(s) (Lange et al., 2012; Loeys et al., 2013; Vansteelandt et al., 2012). For example, for the model $E[Y_{a,M_{a^{*}}}|C]$ = $\beta_{0}+ \beta_{1}a+ \beta_{2}a^{*}+ \beta_{3}C$, the natural direct effect is estimated by $\beta_{1}(a-a^{*})$ and the natural indirect effect by $\beta_{2}(a-a^{*})$.

For the model

$logit\left[ Pr\left( Y_{a, M_{a^{*}}} \right) | C \right]= \beta_{0}+\beta_{1}a+\beta_{2}a^{*}+\beta_{3}a.a^{*}+\beta_{4}C$……………………………………………..….…..Equation 2

the natural direct effect odds ratio can be estimated by $exp [(\beta_{1}+ \beta_{3}a^{*})(a-a^{*})]$ and the natural indirect effect odds ratio by $exp [{(\beta}_{2}+\beta_{3}a)(a-a^{*})$ (Lange et al., 2012). Equation 2 allows for interaction between 𝑎 and $a^{*}$. If this relationship is not of interest to researchers $\beta_{3}$ can be set to zero.

Another major advantage of natural effect models as opposed to the traditional mediation analysis approach is that it yields effects that can be interpreted on the scale of the observed data, and can validly be used for any type of exposure, mediator, or outcome (Vansteelandt et al., 2012).

Natural effect models can be fitted in practice using a weighting-based approach or an imputation-based approach. The weighting-based approach yields consistent natural effect estimates if both the natural effect model and the conditional distribution of the mediator are correctly specified. When dealing with continuous mediators, correct modeling not only demands adequate specification of the mediator's expectation, but also requires additional parametric assumptions on the mediator's conditional density (i.e., the distribution of the error terms) (Steen et al., 2017). Hence, the routine application of the imputation-based approach has been recommended, especially when dealing with continuous mediators, since it avoids reliance on a model for the mediator (Vansteelandt et al., 2012).

**Imputation-based approach for natural effect models**

$Y_{a, M_{a^{*}}}$ is only observable when $a^{*}$ equals 𝑎 and, in addition, 𝑎 corresponds to the observed exposure level *A.* When, $a^{*}$ differs from 𝑎, then $Y_{a, M_{a^{*}}}$ can be predicted as $E(Y|A=a, M,C)$ for $a^{*}$ equalling the observed exposure level, and $M_{a^{*}}$ equalling $M$ among subjects with $a^{*}$ under a suitable model for the outcome. Applying this rationale, imputed datasets can complement the observed dataset, in which the same individuals are evaluated at different exposure levels but corresponding to the observed level of the mediator (Vansteelandt et al., 2012).

**Quantitative intersectionality using three-way decomposition of mediation effects**

In the three-way decomposition of mediated effects, the total indirect effect (natural indirect effect) can be further be separated into a pure indirect effect and a mediated interaction effect (VanderWeele, 2013). This decomposition lends itself to quantitative intersectionality by allowing the effects of the mediator to be evaluated at different levels, while also permitting heterogeneity of mediator effects (Bauer and Scheim, 2019).

To obtain the pure direct, pure indirect, and mediated interaction effects using a traditional (formula-based) three-way decomposition requires using expressions that are confounder(s) or mediator dependent (Bauer and Scheim, 2019; Steen et al., 2017; VanderWeele, 2013). Natural effect models, which (as in Equation 2) model the impact of both confounders and all levels of treatment on the outcome simultaneously, can simplify the application of three-way decomposition methods.

For example, using Equation 2, for a binary outcome, three-way decomposition can be easily approached, such that pure direct effect odds ratio is captured by $exp [(\beta_{1}+ \beta_{3}a^{*})(a-a^{*})]$, pure indirect effect odds ratio by $exp[\left( \beta_{2}+\beta_{3}a^{*} \right)\left( a-a^{*} \right)]$, and mediated interacted effect odds ratio by $exp[\beta_{3}\left( a-a^{*} \right)]$ (Lange et al., 2012)

**Practical application of imputation-based approach of natural effect models**

In our analysis, the exposure is multi-categorical, (i.e., intersectional groups **Group 1**: White HIV-negative, **Group 2**: White living with HIV, **Group 3**: Racialized HIV-negative, **Group 4**: Racialized living with HIV). The mediator is continuous but not distributed normally, i.e., perceived discrimination scores measured on the Everyday Discrimination Scale (Hyman et al., 2019). The outcome is binary, i.e., MHS use in the past 6 months (yes/no). Confounders were age (continuous), having a lifetime chronic mental health condition diagnosed > 6 months ago (yes/no), Canadian citizenship (yes/no), being cisgender (yes/no), and city of enrollment (Montreal, Toronto, or Vancouver).

We conducted our analysis using the *medflex* package in R (Steen et al., 2017). However, we also manually coded for the imputation-based natural effect model (Vansteelandt et al., 2012). We describe this in brief below.

**Imputation step - creating an imputed dataset using simple imputation**

1. A logit model for MHS use conditional on the exposure, mediator, and confounders using the original dataset was fit.

2. A new dataset was created by repeating each observation in the original dataset four times, including two additional variables: $a$ which was equal to the observed exposure (intersectional group) for the first replication and the three other exposure categories for the next three replications, and $a^{*}$ which was equal to the observed exposure for all replications.

3. $Y_{a, M_{a^{*}}}$ was imputed as $Y$for the rows in which $a$ was equal to $a^{*}$ and as the expected probability $P(Y|A=a, M=M_{a^{*}},C)$ for the remaining rows.

**Natural effect model step**

4. A natural effect model of the form specified in Equation 2 was then fitted on the expanded imputed dataset, regressing the imputed outcome on 𝑎, $a^{*}$, and 𝑐. Standard errors and confidence intervals were obtained using bootstrap.

The imputation-based approach for a natural effect model requires that the imputation model be sufficiently rich to prevent incoherence (uncongeniality) with the natural effect model (Vansteelandt et al., 2012). In our analysis, the same set of variables were used for both the imputation and natural effect models. The additional inclusion of education, income and employment in the imputation model did not change effect estimates of the natural effect model.

Note that while the imputation-based approach generates potential outcomes assuming counterfactual exposures, our results are interpreted through the perspective of intervening on the mediator. This is done because, in actuality, racial identity and HIV seropositivity are not modifiable (VanderWeele and Robinson, 2014).

**Supplementary Figure 2: Timeline for the exposure, mediator, outcome, and confounders**


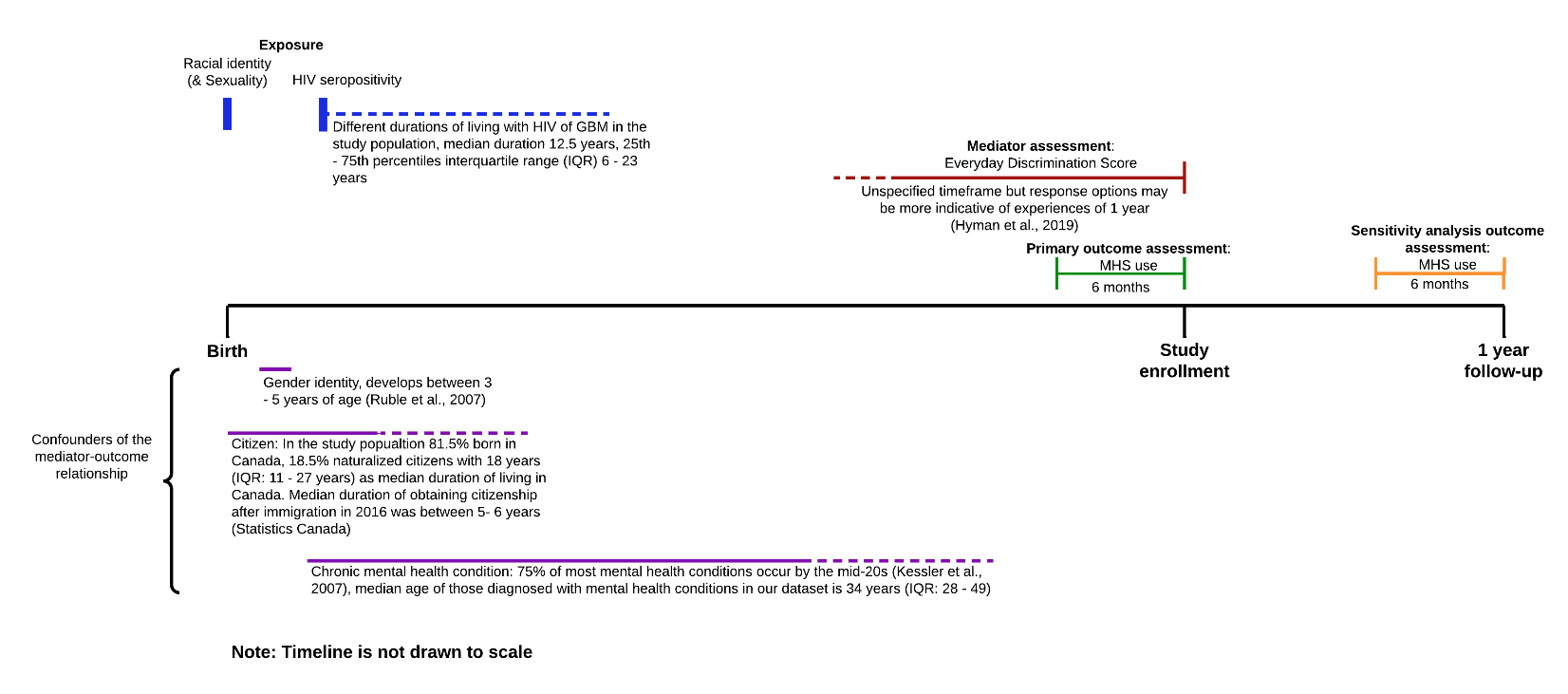


| GBM: Gay, bisexual and other men who have sex with men; MHS: Mental Health Services; IQR: 25^th^ to 75^th^ percentiles interquartile range  We describe confounders of the mediator-outcome relationship and not confounders in general, given that our findings are interpreted based on potential interventions on the mediator and not on the exposure (mentioned in the main manuscript) (VanderWeele and Robinson, 2014). Other confounders not included in the figure are age and city – these were measured at study enrollment.  Dashed lines (of any color) indicate that the time duration for events is not definitively defined with respect to birth and study enrollment. For example, in our study population, the confounder - living with a chronic mental health condition is defined as those diagnosed with a condition> 6 months ago. The median age of those diagnosed is 34 years (IQR: 28 -49), and previous research indicates that 75% of most mental health conditions occur by an individual’s mid-20s (Kessler et al., 2007). Thus, while different GBM in our cohort may be living with mental health condition(s) for different durations, we assume many would be living with condition(s) for durations prior to the time frames of the mediator or outcome assessment. |
| --- |

| **Supplementary Table 1: Log odds estimates for the entire natural effect model including confounders** | | |
| --- | --- | --- |
| **Comparator exposure group**: Group 1 (White HIV-negative GBM) | | |
| **Mediator:** Standardized Everyday Discrimination Scale scores | | |
|  | **Log odds** | **95% Confidence Interval^a^** |
| Intercept | -0.0016 | -0.4713, 0.4693 |
| PDE Group 2 | 0.5216 | 0.2363, 0.8063 |
| PIE Group 2 | 0.0159 | -0.0164, 0.0469 |
| MIE Group 2 | -0.0008 | -0.0188, 0.0191 |
| TIE Group 2 | 0.0151 | -0.0180, 0.0483 |
| PDE Group 3 | -0.1754 | -0.4227, 0.0885 |
| PIE Group 3 | 0.0901 | 0.0190, 0.1585 |
| MIE Group 3 | 0.0352 | -0.0702, 0.1403 |
| TIE Group 3 | 0.1253 | 0.0389, 0.2117 |
| PDE Group 4 | 0.2051 | -0.3082, 0.7667 |
| PIE Group 4 | 0.0805 | -0.0056, 0.1604 |
| MIE Group 4 | -0.0783 | -0.2739, 0.1205 |
| TIE Group 4 | 0.0022 | -0.0020, 0.0063 |
| **Confounders** | | |
| **Age** | -0.0117 | -0.0194, -0.0039 |
| **City**  Montreal  Toronto  Vancouver | Reference  0.3194  0.2003 | -  0.0690, 0.5609  -0.0226, 0.4141 |
| **Canadian citizenship**  Yes  No | Reference  -0.5479 | -  -0.8105, -0.2733 |
| **Chronic mental health condition**  Yes  No | 0.5124  Reference | 0.3144, 0.7070  - |
| **Cisgender**  Yes  No | -0.7669  Reference | -1.1477, -0.3905  - |
| **Group 2**: White living with HIV, **Group 3**: Racialized HIV-negative, **Group 4**: Racialized living with HIV  ^a^Normal approximation bootstrap confidence intervals based on 1000 bootstrap samples  **PDE:** Pure direct effect, **PIE:** Pure indirect effect, **MIE:** Mediated interaction effect, **TIE:** Total indirect effect obtained by adding the PIE and MIE for each respective group.  Note that using the TIE (without decomposing it to PIE and MIE) assumes that there is no interaction between the exposure and the mediator – i.e., the effect of the mediator on the outcome for all exposure groups is comparable. While this may be mathematically plausible for a given dataset (e.g., in our case where the MIEs are not statistically significant), it is in contradiction to an intersectionality-based theoretical approach which emphasizes the importance of historical and social contexts in shaping experiences of people located at different social intersections. It is also important to consider that interaction effects do frequently suffer from low statistical power and that the absence of statistical significance may be indicative a lack of power rather than a lack of effect. | | |

The log odds for the estimates of the natural effect model are presented in Supplementary Table 1, including for the confounders. Since our analyses use a causal model, confounder log odds estimates are not used or presented as primary results. Doing so may result in a Table 2 fallacy (Westreich and Greenland, 2013).

To use RDS weights in the natural effect models when data was combined across the three cities, we undertook the following procedures: First, a sum of the RDS weights was obtained separately for each city. Then, each individual participant weight for a given city was divided by the total RDS weight of that city. These were then combined across the three cities and used in the natural effect model as probability weights. Normalizing the weights by city in this way ensures that the relative probability of being sampled by city is maintained while avoiding undue influence of a given city if network size varies meaningfully across cities.

| **Supplementary Table 2: Odds ratios for MHS use in the past 6 months across different intersectional categories, accounting for normalized RDS weights** | | | |
| --- | --- | --- | --- |
|  | **Reference**: Group 1  **Comparison**: Group 2 | **Reference**: Group 1  **Comparison**: Group 3 | **Reference**: Group 1  **Comparison**: Group 4 |
| **Total effect** | 1.71 (1.28, 2.29) | 0.95 (0.74, 1.22) | 1.23 (0.72, 2.09) |
| **Pure direct effect** | 1.68 (1.26, 2.25) | 0.84 (0.65, 1.10) | 1.23 (0.71, 2.14) |
| **Pure indirect effect** | 1.02 (0.98, 1.05) | 1.09 (1.02, 1.17) | 1.08 (1.00, 1.17) |
| **Mediated interaction effect** | 1.00 (0.98, 1.02) | 1.04 (0.93, 1.15) | 0.92 (0.76, 1.13) |
| **Confounders adjusted for in the model**: age, city, having a chronic mental health condition, Canadian citizenship, and cisgender status | | | |

| **Supplementary Table 3: Odds ratios for MHS use in the past 6 months across different intersectional categories, with additional confounders for socioeconomic status i.e., income, education, and employment** | | | |
| --- | --- | --- | --- |
|  | **Reference**: Group 1  **Comparison**: Group 2 | **Reference**: Group 1  **Comparison**: Group 3 | **Reference**: Group 1  **Comparison**: Group 4 |
| **Total effect** | 1.77 (1.31, 2.39) | 0.96 (0.75, 1.22) | 1.28 (0.77, 2.13) |
| **Pure direct effect** | 1.76 (1.30, 2.38) | 0.84 (0.65, 1.10) | 1.29 (0.77, 2.21) |
| **Pure indirect effect** | 1.00 (0.97, 1.04) | 1.10 (1.02, 1.18) | 1.07 (0.99, 1.17) |
| **Mediated interaction effect** | 1.00 (0.98, 1.02) | 1.03 (0.92, 1.15) | 0.92 (0.76, 1.11) |
| **Confounders include** age, city, having a chronic mental health condition, Canadian citizenship, and cisgender status, income, education, and employment. Income, education, and employment were used as categorical variables as specified in Table 1 (main manuscript). | | | |

The Medical Outcomes Study (MOS) Social Support Survey is a 19-item instrument that measures the availability of people to provide support if needed such as emotional support and tangible help (Sherbourne and Stewart, 1991). Each item is scored on a 5-point Likert scale. Total scores range between 19 and 95. The scale showed good internal consistency across all three cities (Cronbach’s α > 0.95).

| **Supplementary Table 4a: Mean scores of the MOS Social Support Survey across intersectional groups** | |
| --- | --- |
|  | **Mean scores (SD)** |
| Group 1 | 73.8 (16.1) |
| Group 2 | 67.6 (19.8) |
| Group 3 | 69.5 (17.9) |
| Group 4 | 65.1 (21.5) |

| **Supplementary Table 4b: Odds ratios for MHS use in the past 6 months across different intersectional categories with an additional confounder for social support scores measured on the MOS Social Support Survey Instrument** | | | |
| --- | --- | --- | --- |
|  | **Reference**: Group 1  **Comparison**: Group 2 | **Reference**: Group 1  **Comparison**: Group 3 | **Reference**: Group 1  **Comparison**: Group 4 |
| **Total effect** | 1.71 (1.28, 2.28) | 0.96 (0.74, 1.24) | 1.20 (0.70, 2.05) |
| **Pure direct effect** | 1.68 (1.26, 2.24) | 0.83 (0.64, 1.09) | 1.21 (0.70, 2.14) |
| **Pure indirect effect** | 1.02 (0.98, 1.05) | 1.10 (1.02, 1.19) | 1.09 (1.00, 1.19) |
| **Mediated interaction effect** | 1.00 (0.97, 1.02) | 1.04 (0.93, 1.16) | 0.90 (0.74, 1.10) |
| **Confounders adjusted for in the model**: age, city, having a chronic mental health condition, Canadian citizenship, and cisgender status, mean scores of the MOS Social Support Survey Instrument measured at baseline | | | |

The Connor-Davidson Resilience Scale-2 is a brief 2-item measure of resilience or how well one is equipped to bounce back after stressful events, tragedy, or trauma (Vaishnavi et al., 2007). Each item is scored on a 5-point Likert scale. Total scores range between 0 and 8. The Cronbach’s α for Montreal was 0.75, for Toronto was 0.94 and Vancouver 0.84.

| **Supplementary Table 5a: Mean scores on the Connor-Davidson Resilience Scale-2 across intersectional group** | |
| --- | --- |
|  | **Mean (SD)** |
| Group 1 | 6.3 (1.5) |
| Group 2 | 6.4 (1.7) |
| Group 3 | 6.0 (1.8) |
| Group 4 | 6.2 (1.7) |

| **Supplementary Table 5b:** **Odds ratios for MHS use in the past 6 months across different intersectional categories with an additional confounder for resilience measured on the Connor Davidson Scale-2 (mean scores)** | | | |
| --- | --- | --- | --- |
|  | **Reference**: Group 1  **Comparison**: Group 2 | **Reference**: Group 1  **Comparison**: Group 3 | **Reference**: Group 1  **Comparison**: Group 4 |
| **Total effect** | 1.70 (1.27, 2.26) | 0.94 (0.73, 1.20) | 1.27 (0.75, 2.16) |
| **Pure direct effect** | 1.67 (1.24, 2.20) | 0.83 (0.64, 1.08) | 1.25 (0.72, 2.16) |
| **Pure indirect effect** | 1.01 (0.98, 1.04) | 1.08 (1.02, 1.16) | 1.07 (1.00, 1.14) |
| **Mediated interaction effect** | 1.00 (0.98, 1.02) | 1.03 (0.93, 1.15) | 0.95 (0.78, 1.14) |
| **Confounders adjusted for in the model**: age, city, having a chronic mental health condition, Canadian citizenship, and cisgender status, mean scores measured on the Connor Davidson Resilience Scale-2 measured at baseline | | | |

| **Supplementary Table 6: Odds ratios for MHS use in the past 6 months across different intersectional categories, lagging the outcome by 1 year and accounting for loss to follow-up by using inverse probability weights** | | | |
| --- | --- | --- | --- |
|  | **Reference**: Group 1  **Comparison**: Group 2 | **Reference**: Group 1  **Comparison**: Group 3 | **Reference**: Group 1  **Comparison**: Group 4 |
| **Total effect** | 1.26 (0.89, 1.77) | 0.98 (0.74, 1.29) | 1.18 (0.64, 2.16) |
| **Pure direct effect** | 1.25 (0.87, 1.75) | 0.81 (0.60, 1.10) | 1.11 (0.62, 2.06) |
| **Pure indirect effect** | 1.02 (0.98, 1.05) | 1.09 (1.01, 1.18) | 1.08 (0.98, 1.18) |
| **Mediated interaction effect** | 0.99 (0.95, 1.03) | 1.11 (0.98, 1.26) | 0.98 (0.75, 1.29) |
| **Confounders adjusted for in the model**: age, city, having a chronic mental health condition, Canadian citizenship, and cisgender status measured at baseline | | | |

Inverse probability weights were created by fitting a logistic regression model of loss to follow-up status in the first year of follow-up (yes/no) on variables that were identified to potentially affect retention but also associated with the outcome (Willems et al., 2018). These were age, cisgender identity, HIV status, education, income, Canadian citizenship, racial identity (racialized or not), discrimination (EDS mean scores), living with a chronic mental health condition and city. Variables were categorized as indicated in Table 1 of the main manuscript.

The inverse probability of not being lost to follow up was then obtained for each participant. These were used as weights in the natural effect models. Thus, participants who had a higher probability of being lost were weighted up and those who had lower probability of being lost were weighted down. This accounts for the selection bias that may be produced by only including participants that were retained.

| **Supplementary Table 7: Comparison of selected socioeconomic factors between intersectional groups** | | | | |
| --- | --- | --- | --- | --- |
|  | **White HIV- negative**  **(n=1376)** | **White living with HIV**  **(n=327)** | **Racialized HIV-negative**  **(n=577)** | **Racialized living with HIV**  **(n=91)** |
|  | **Unadjusted n (%)** | **Unadjusted n (%)** | **Unadjusted n (%)** | **Unadjusted n (%)** |
| **Mean age (SD)** | 36 (12.8) | 48.0 (11.3) | 31.7 (9.3) | 41.4 (11.8) |
| **Education** **≤ High school** | 212 (15.4) | 77 (23.5) | 62 (10.8) | 22 (24.2) |
| **Annual Income < CAD 30, 000** | 627 (45.6) | 201 (61.5) | 319 (55.3) | 61 (67.0) |
| **Unemployed** | 323 (23.5) | 150 (45.8) | 145 (25.1) | 43 (47.3) |
| **Immigrant** | 203 (14.7) | 18 (5.5) | 223 (38.6) | 26 (28.6) |
| **No primary health care provider** | 444 (32.3) | 14 (4.3) | 210 (36.4) | 6 (6.6) |
| **No medical insurance** | 403 (29.3) | 80 (24.5) | 181 (31.4) | 21 (23.1) |
| Here, select socioeconomic factors (e.g., lower income, higher unemployment) are thought of as ***structural*** inequity factors that may increase chronic stress, particularly among GBM living with HIV. This ‘additional’ inequity among GBM living with HIV must be contextualized in the background of pre-existing higher unemployment and lower income among Canadian GBM (compared to heterosexual men) in general (Kinitz et al., 2023; Waite et al., 2019). | | | | |

**Timeline of events involving GBM, racialized groups, and people living with HIV 1980-2023 with a focus on events at the national level or in Montreal, Toronto, and Vancouver**

(The timeline below is a brief, non-comprehensive list of events. We provide these for interested readers to appreciate the historical context that frames our analysis)

**1981:** Police raid four gay bathhouses under ‘Operation Soap’ on February 5 in Toronto and arrest 300 patrons. The next night 3,000 people march in downtown Toronto. This is considered Canada’s Stonewall (Queer Events Editorial Staff, 2019).

**1982:** First case of HIV in Canada is detected (CBC News, 2018). The Canadian Psychiatric Association removes homosexuality from its list of mental disorders. It remains in Alberta’s guide to mental-health disorders till 2010 (CBC News, 2010).

**1985**: Kenneth Zeller a 40-year-old librarian is murdered in a homophobic hate crime in Toronto’s High Park. This spurs the Toronto District School Board to implement one of Canada’s first programs to combat anti-gay discrimination and violence (Queer Events, 2021).

**1987**: Anthony Griffin, a 19-year-old unarmed Black man is fatally shot in Montreal on November 11, 1987, after initially resisting but later cooperating with his arrest. On November 21, two thousand people attend a march to protest the shooting. Another protest of approximately 1000 people takes place on February 27, 1988. The police officer involved is acquitted of all charges (Nerestant et al., 2017).

**1988**: Prime Minister Brian Mulroney provides a formal apology for sending 21,000 Japanese Canadians to internment camps in 1942 (McRae, 2017a).

**1988:** AIDS Action Now is formed in Toronto. They burn the effigy of Federal Health Minister, Jake Epp who avoids saying the word, ‘AIDS’. He is replaced by Perrin Beatty (Allen, 2020).

**1988:** Police shoot and kill Michael Wade Lawson, a 17-year-old Black teenager, after finding him in a stolen car (Rosella, 2015), and Lester Donaldson, a 44-year-old Black man during a confrontation in his rooming house (Falconers LLP, 1989). Police officers in both cases are acquitted. The Ontario government creates the Race Relations and Policing Task Force in response (Ontario Human Rights Commission, 2018).

**1989**: Joe Rose, a 24-year-old gay activist living with HIV is stabbed to death in a homophobic hate crime on March 19, 1989, in Montreal (MacFarlane, 2019). Outrage over his death prompts the formation of the activist group Réaction SIDA. Its members together with those from ACT UP and AIDS Action Now storm the International AIDS Society Conference in July. Organizers and attendees of the conference are accused of careerism, using the experiences and data of people living with AIDS while excluding them from the conference. Prime Minister Brian Mulroney, after five years in office, makes his first public remarks on AIDS at the conference (Couturier, 2022).

**1990:**  Montreal police raid an after-hours venue called the Sex Garage on July 15, 1990, force four hundred LGBTQ individuals into the street and attack them with batons, injuring some severely. A 250-person strong protest in front of the police station the next day leads to violent retaliation by the police and the arrest of nearly 50 protestors, who are further abused in police custody (Queer Events Editorial Staff, 2019).

**1990:** The term Two-spirit (*niizh manidoowag*) is coined at the third annual Native American/First Nations Gay and Lesbian Conference in Winnipeg. The term allows Indigenous LGBTQ+ individuals to reject western views of sexuality and gender (Filice, 2015).

**1950s to mid-1990’s:** LGBTQ individuals in the Canadian Armed Forces, the RCMP and the federal public service are systematically discriminated against, harassed, and fired as a matter of policy and sanctioned governmental practice, in what is known as the ‘LGBTQ Purge’. About 9000 individuals are estimated to have lost their jobs, some imprisoned for their sexual orientation, and several believed to have committed suicide (LGBT Purge Fund, 2023). Prime Minister Justin Trudeau offers an official apology in 2017 (Harris, 2017).

**1992:** Two leaders of the KKK in Montreal, Michel Larocque and Alain Roy, and 36 Skinheads are arrested in Montreal while trying to set fire to a building (Noël, 2018).

**1993:** The murders of 14 gay men in Montreal remain unsolved and the police refuses to acknowledge the targeting of gay men. Victims include Warren Ealing, an Anglican priest and Roland Gagnier, a Catholic priest. Andrew Hutchinson, the Anglican Bishop of Montreal, acknowledges that Ealing is targeted for his sexual identity. Roger LeClerc, a gay activist threatens to publicly ‘out’ gay politicians and lawmakers in Quebec in response to governmental inaction. Yves Lalonde, a gay man is killed by four neo-Nazi teenagers. The Quebec government establishes an advisory commission on discrimination against gay individuals (CBC Listen, 2022).

**1990 – 2004**: An estimated 120 anti-gay related homicides and 350 acts of homophobic violence are committed in Canada (Janoff, 2005).

**2001**: Aaron Webster, a 42-year-old gay man is murdered in Vancouver. On November 18, 2001, over 3,000 people come together for a march and vigil protesting anti-gay violence (Adach and Eifling, 2011).

**2005:** Canada becomes the fourth country in the world and the first outside Europe to officially sanction same-sex marriage (Cotler, 2015).

**2006:** Prime Minister Stephen Harper offers an official apology to Chinese Canadians for the Head Tax (a fee for Chinese persons entering Canada levied by the government from 1885 to 1923) and the subsequent exclusion of Chinese immigrants from 1923 to 1947 (Yu, 2019).

**2008 - 2022:** David Popescu, an independent politician responds to a question about same sex-marriage by stating that, “homosexuals should be executed”. He calls for the execution of Kathleen Wynne, Ontario’s former premier who is gay, in 2018. He is sentenced to jail twice for anti-gay hate crimes. He runs as an independent candidate from Sudbury, Ontario in June 2022 (Sudbury.com Staff, 2021).

**2009:** Ritchie Dowrey, a 62-year-old gay man is physically attacked in an anti-gay hate crime by Shawn Woodward in Vancouver, leaving him with permanent paraplegia and memory loss (CBC News, 2015).

**2010 – 2017:** Bruce McArthur murders eight gay men in Toronto. Six of his victims were immigrants from Afghanistan, Sri Lanka, Iran, and Turkey (BBC, 2019).

**2011:** Jamie Hubbley, the son of Ottawa city councillor commits suicide following anti-gay bullying at school. This leads to the Legislative Assembly of Ontario passing stiffer penalties for bullying in schools in 2012 (CBC News, 2011).

**2011 – 2012**: An increase in homophobic violent incidents in Montreal’s gay village prompts Montreal’s non-profit Gai Écoute (now Interligne) to launch the Acts of Homophobia registry (Newsdesk, 2012).

**2015**: The Truth and Reconciliation Committee presents its final report, including 94 calls to action to further reconciliation between Canadians and Indigenous Peoples (Mas, 2015). According to Indigenous Watchdog (2023), a federally registered non-profit, as of September 2023, 14% of those action to calls have been completed.

**2016:** Prime Minister Justin Trudeau apologizes for the SS Komagata Maru incident of 1914, in which 376 passengers from India aboard the ship were not allowed to enter Vancouver (McRae, 2017b).

**2016**: A Canadian Institute of Health Social Research Centre report finds that Canadian mainstream newspapers feature Black immigrant men in articles four times more often than would be warranted based on the proportion of defendants they represent in cases of HIV criminalization (Mykhalovskiy et al., 2016)

**2017**: HIV Annual Report for British Columbia shows that the proportion of new HIV cases is highest among gay, bisexual and men who have sex with men (GBM). Of these, 7.9% are among Indigenous men (up from 5.5% in 2008) and 15% among Asian men (up from 11.6% in 2008) (BC Centre for Disease Control, 2019).

**2019**: A Canadian Centre for Policy Alternatives report using 2016 census data shows the racialization of income inequality in Canada (20.8% of racialized families were below the low-income measure compared to 12.2% for non-racialized families. Those identifying as Arab, West Asian, Korean had nearly three times higher poverty rates compared to non-racialized families. Relative to non-racialized immigrant men, racialized immigrant men earned 71 cents per $1, non-racialized immigrant women earned 66 cents per $1, and racialized immigrant women earned 52 cents per $1) (Block et al., 2019).

**2020:** Canadians of Asian ethnicity find themselves the targets of widespread racism and accusations of complicity in the release of the coronavirus**.** In Vancouver, anti-Asian hate crimes increase by 717% between 2019 and 2020 (Zussman, 2021).

**2020:** Joyce Echaquan, a 37-year-old Atikamekv woman dies in a Quebec hospital. Before her death, she faces racist taunts by the health care staff (APTN National News, 2020). British Columbia publishes, “In Plain Sight” (Government of British Columbia, 2020), in which a survey of 2,780 Indigenous Peoples individuals highlights widespread stereotyping and racism in health care.

**2020:**  The HIV Annual Report for Ontario shows that the proportion of new HIV cases is highest among GBM. Proportions among Black GBM increased from 12.5% in 2016 to 17.1% in 2020, and for Latino/x GBM increased from 10.2% in 2016 to 15.6% in 2020 (Ontario HIV Epidemiology and Surveillance Initiative, 2022).

**2021:** Conversion therapy is banned in Canada (Treisman, 2021). However, it is still actively being practiced under the guise of medical and mental health care, or behind religious authorities, according to Fondation Émergence (2023), a non-profit based in Quebec.

**2022:** The Canadian government removes deferral period for men who have sex with men (MSM) to donate blood, if they test negative for HIV. Until then Canada had deferral periods for MSM to donate blood (5-years from last sexual intercourse till 2016, 1-year till 2019, 3-months till Sep 2022) ( (Branch, 2022).

**2022**: The Government of Canada launches a public consultation to reform the criminal law regarding HIV non-disclosure (Government of Canada, 2023b). There have been at least 224 prosecutions to date in Canada for HIV non-disclosure, most of them as aggravated sexual assault. The 70% conviction rate in HIV non-disclosure cases is higher than the rate for other sexual assault cases (one of the highest in the world) and prison sentences are more than double the average sentence for sexual assault (Csete et al., 2023).

**2022**: A report by Public Safety Canada (2023) shows 26.1% of all prisoners being Indigenous (Indigenous Peoples constitute 5.0% of Canada’s population) and 8.1% being Black (Black individuals account for 3.5% of Canada’s population).

**2022 to present:** Drag queen story hours at libraries across Canada are targeted by anti-gay activists (McGinn, 2023).

**2023**: A report by Statistics Canada shows that between 2020 and 2021, police-reported hate crimes motivated by sexual orientation increase by 64%, hate crimes motivated by race or ethnicity increase by 6% (after rising by 83% between 2019-2020). Hate crimes against East and Southeast Asians increase by 16%, South Asian 21%, and West Asian & Arab by 46% in the same period (Statistics Canada, 2023).

**References**

**Adach K and Eifling S** (2011) *Ten Years After Aaron Webster’s Death, What’s Changed?* Available at: https://thetyee.ca/News/2011/11/17/Homophobic-Hate-Crimes/ (accessed 18 March).

**Alberta Civil Liberties Research Centre** (2021) *Racialization*. Available at: https://www.aclrc.com/racialization (accessed 01 May).

**Allen K** (2020) *AIDS 1988: lighting a fire under Health Minister Epp*. Available at: https://calgarygayhistory.ca/2020/04/10/aids-1988-lighting-a-fire-under-health-minister-epp/ (accessed 20 March).

**American Psychological Association** (2023) *APA Dictionary of Psychology: racism*. Available at: https://dictionary.apa.org/racism?_ga=2.96995095.900644839.1684184268-2086010642.1674612759 (accessed 02 May).

**APTN National News** (2020) *Joyce Echaquan*. Available at: https://www.aptnnews.ca/topic/joyce-echaquan/ (accessed 30 March).

**Bauer GR and Scheim AI** (2019) Advancing quantitative intersectionality research methods: Intracategorical and intercategorical approaches to shared and differential constructs. *Social Science & Medicine* 226: 260-262.

**BBC** (2019) *Bruce McArthur: Canadian landscaper admits eight murders*. Available at: https://www.bbc.com/news/world-us-canada-47047394 (accessed 19 March).

**BC Centre for Disease Control** (2019) *HIV in Bristish Columbia: Annual Surveillance Report 2017. Retrieved from http://www.bccdc.ca/health-professionals/data-reports/hiv-aids-reports*. (accessed 1 June).

**Block S, Galabuzi G-E and Tranjan R** (2019) *Canada's Colour Coded Income Inequality*. Available at: https://policyalternatives.ca/sites/default/files/uploads/publications/National%20Office/2019/12/Canada%27s%20Colour%20Coded%20Income%20Inequality.pdf (accessed 2 May).

**Branch H** (2022) *CIHR Canadian HIV Trials Network: Blood donation criteria due to change for men who have sex with men*. Available at: https://www.hivnet.ubc.ca/news/2022/08/blood-donation-criteria-due-to-change-for-men-who-have-sex-with-men/ (accessed 10 April).

**CBC Listen** (2022) *The Village: The Montreal Murders*. Available at: https://www.cbc.ca/radio/podcastnews/the-village-the-montreal-murders-transcripts-listen-1.6479960 (accessed 19 March).

**CBC News** (2010) *Homosexuality pulled from Alberta disorders guide*. Available at: https://www.cbc.ca/news/canada/edmonton/homosexuality-pulled-from-alberta-disorders-guide-1.939190 (accessed 20 March).

**CBC News** (2011) *Gay Ottawa teen who killed himself was bullied*. Available at: https://www.cbc.ca/news/canada/ottawa/gay-ottawa-teen-who-killed-himself-was-bullied-1.1009474 (accessed 11 May).

**CBC News** (2015) *Ritch Dowrey, gay-bashing victim at Fountainhead Pub in 2009, dead*. Available at: https://www.cbc.ca/news/canada/british-columbia/ritch-dowrey-gay-bashing-victim-at-fountainhead-pub-in-2009-dead-1.2942640 (accessed 21 March).

**CBC News** (2018) *Phobia about HIV still exists over 30 years after 1st AIDS case in Canada, activists say*. Available at: https://www.cbc.ca/news/canada/toronto/aids-activism-pride-toronto-35-years-1.4719204 (accessed 19 April).

**Centers for Disease Control and Prevention** (2021) *HIV Stigma and Discrimination: What is HIV stigma?* Available at: https://www.cdc.gov/hiv/basics/hiv-stigma/index.html#:~:text=HIV%20discrimination%20is%20the%20act,with%20someone%20living%20with%20HIV (accessed 02 May).

**Cotler I** (2015) *How Canada led the way on same-sex marriage*. Available at: https://www.thestar.com/opinion/contributors/how-canada-led-the-way-on-same-sex-marriage/article_875ec06c-4268-590f-97a1-e24303778047.html (accessed 19 March).

**Couturier C** (2022) *Années 1980: Montréal au coeur de la lutte contre le sida*. Available at: https://www.ledevoir.com/societe/science/755670/les-annees-1980-montreal-au-coeur-de-la-lutte-contre-le-sida (accessed 18 March).

**Csete J, Elliott R and Bernard EJ** (2023) So many harms, so little benefit: a global review of the history and harms of HIV criminalisation. *Lancet HIV* 10(1): e52-e61.

**Doebler S** (2015) Relationships between Religion and Two Forms of Homonegativity in Europe--A Multilevel Analysis of Effects of Believing, Belonging and Religious Practice. *PLoS One* 10(8): e0133538.

**Falconers LLP** (1989) *Lester Donaldson Inquest*. Available at: https://falconers.ca/casestudy/lester-donaldson-inquest/ (accessed 10 March).

**Filice M** (2015) *The Canadian Encylopedia: Two-Spirit*. Available at: https://www.thecanadianencyclopedia.ca/en/article/two-spirit (accessed 19 March).

**Fondation Émergence** (2023) *En finir avec les thérapies de conversion*. Available at: https://www.fondationemergence.org/therapie-de-conversion (accessed 20 April).

**Fudge Schormans A** (2014) Stigmatization. In: Michalos AC (ed) *Encyclopedia of Quality of Life and Well-Being Research*, pp.6336-6341. Dordrecht: Springer Netherlands.

**Government of British Columbia** (2020) *In Plain Sight: Addressing Indigenous-specific Racism and Discrimination in B.C. Health Care*. Available at: https://engage.gov.bc.ca/app/uploads/sites/613/2020/11/In-Plain-Sight-Full-Report-2020.pdf (accessed 1 June).

**Government of Canada** (2023a) *Anti-racism lexicon*. Available at: https://www.canada.ca/en/department-national-defence/services/systemic-racism-discrimination/anti-racism-toolkit/anti-racism-lexicon.html#toc17 (accessed 20 April).

**Government of Canada** (2023b) *Reforming the criminal law regarding HIV non-disclosure – Online Public Consultation*. Available at: https://www.justice.gc.ca/eng/cons/hiv-vih/index.html (accessed 20 April).

**Harris K** (2017) *'Our collective shame': Trudeau delivers historic apology to LGBT Canadians*. Available at: https://www.cbc.ca/news/politics/homosexual-offences-exunge-records-1.4422546 (accessed 19 May).

**Hyman I, O'Campo P, Ansara DL, Siddiqi A, Forte T, Smylie J, Mahabir DF and McKenzie K** (2019) Prevalence and Predictors of Everyday Discrimination in Canada: Findings from the Canadian Community Health Survey. Wellesly Institute.

**Indigenous Watchdog** (2023) *Calls to Action: The Truth and Reconciliation Commission of Canada*. Available at: https://www.indigenouswatchdog.org/calls-to-action/ (accessed April 27).

**Janoff DV** (2005) *Pink Blood : Homophobic Violence in Canada.* University of Toronto Press.

**Kessler RC, Amminger GP, Aguilar-Gaxiola S, Alonso J, Lee S and Ustün TB** (2007) Age of onset of mental disorders: a review of recent literature*. Current Opinion in Psychiatry* 20(4), 359-364. <https://doi.org/10.1097/YCO.0b013e32816ebc8c>.

**Kinitz DJ, Gould WA, Shahidi FV, MacEachen E, Mitchell C, Craig-Venturi D and Ross LE** (2023) Addressing knowledge gaps about skills of 2SLGBTQ+ people in Canada: A scoping review and qualitative inquiry. A report for Employment and Social Development Canada. Retrieved from: https://lgbtqhealth.ca/projects/foundationaltransferableskills.php.

**Lange T, Vansteelandt S and Bekaert M** (2012) A simple unified approach for estimating natural direct and indirect effects. *American Journal of Epidemiology* 176(3): 190-195.

**Lazarus RS and Folkman S** (1984) *Stress, appraisal, and coping.* New York: Springer Publishing Company.

**LGBT Purge Fund** (2023) *LGBT Purge Fund: Commemorate. Educate. Advance*. Available at: https://lgbtpurgefund.com/ (accessed 19 March).

**Loeys T, Moerkerke B, De Smet O, Buysse A, Steen J and Vansteelandt S** (2013) Flexible Mediation Analysis in the Presence of Nonlinear Relations: Beyond the Mediation Formula. *Multivariate Behavioral Research* 48(6), 871-894. https://doi.org/10.1080/00273171.2013.832132.

**MacFarlane J** (2019) *30 years later, Montreal's gay community reflects on the murder of Joe Rose*. Available at: https://www.cbc.ca/news/canada/montreal/joe-rose-murder-30-years-1.5062744 (accessed 19 April).

**Major B, Dovidio JF and Link BG** (2017) The Oxford handbook of stigma, discrimination, and health. New York, N.Y.: Oxford University Press.

**Mas S** (2015) *Truth and Reconciliation offers 94 'calls to action'*. Available at: https://www.cbc.ca/news/politics/truth-and-reconciliation-94-calls-to-action-1.3362258 (accessed 10 May).

**McGinn D** (2023) *Anti-gay activists target children’s libraries and drag queen story hours*. Available at: https://www.theglobeandmail.com/canada/article-anti-gay-activists-target-childrens-libraries-and-drag-queen-story/ (accessed 12 April).

**McRae M** (2017a) *Japanese Canadian internment and the struggle for redress*. Available at: https://humanrights.ca/story/japanese-canadian-internment-and-struggle-redress (accessed 18 March).

**McRae M** (2017b) *The story of the Komagata Maru*. Available at: https://humanrights.ca/story/story-komagata-maru (accessed 18 March).

**Mykhalovskiy E, Hastings C, Sanders T, Hayman M and Bisaillon L** (2016) 'Callous, Cold and Deliberately Duplicitous': Racialization, immigration and the Representation of HIV Criminalization in Canadian Mainstream Newspapers. Available at SSRN: https://ssrn.com/abstract=2874409 or <http://dx.doi.org/10.2139/ssrn.2874409>.

**Nerestant A, Codrington D and Leader C** (2017) *A 'senseless' death, but have things changed? Remembering police shooting victim Anthony Griffin*. Available at: https://www.cbc.ca/news/canada/montreal/anthony-griffin-30-year-anniversary-1.4387950 (accessed 24 March).

**Newsdesk** (2012) *Quebec Creates Registry to Track homophobia*. Available at: https://outnewsglobal.com/quebec-creates-registry-to-track-homophobia/ (accessed 19 May).

**Noël B** (2018) *Why Montreal Was a Hotbed for Neo-Nazis in the 90s*. Available at: https://www.vice.com/en/article/qvwav7/why-montreal-was-a-hotbed-for-neo-nazis-in-the-90s (accessed 19 March).

**Ontario HIV Epidemiology and Surveillance Initiative** (2022) *HIV diagnoses in Ontario, 2020*. Available at: https://www.ohesi.ca/wp-content/uploads/2022/08/HIV-diagnoses-in-Ontario-2020-REPORT-FINAL-1.pdf (accessed 2 June).

**Ontario Human Rights Commission** (2018) *Timeline of racial discrimination and racial profiling of Black persons by the Toronto Police Service, and OHRC initiatives related to the Toronto Police*. Available at: https://www.ohrc.on.ca/en/book/export/html/23851 (accessed 19 March).

**Public Safety Canada** (2023) *Corrections and Conditional Release Statistical Overview: 2021 Annual Report*. Available at: https://www.publicsafety.gc.ca/cnt/rsrcs/pblctns/ccrso-2021/ccrso-2021-en.pdf (accessed 10 May).

**Queer Events** (2021) *Canadian Queer History: The Pursuit of LGBT2Q+ Acceptance*. Available at: https://www.queerevents.ca/queer-history/canadian-history-timeline (accessed 10 April).

**Queer Events Editorial Staff** (2019) *The Tipping Point: Four Key Protests in Canadian LGBT2Q+ History*. Available at: https://www.queerevents.ca/queer-history/articles/tipping-points/key-protest-lgbt-history (accessed 10 April).

**Rosella L** (2015) *50TH ANNIVERSARY: Black teen killed by two white cops in 1988*. Available at: https://www.mississauga.com/news/50th-anniversary-black-teen-killed-by-two-white-cops-in-1988/article_c9675649-687b-503e-a4b1-c756526a7a6d.html (accessed 10 April).

**Ruble DN, Taylor LJ, Cyphers L, Greulich FK, Lurye LE and Shrout PE** (2007) The role of gender constancy in early gender development. *Child Development* 78(4), 1121-1136. <https://doi.org/10.1111/j.1467-8624.2007.01056.x>.

**Sherbourne CD and Stewart AL** (1991) The MOS social support survey. *Social Science & Medicine* 32(6): 705-714.

**Statistics Canada** (2019) *Trends in the Citizenship Rate Among New Immigrants to Canada*. Available at: https://www150.statcan.gc.ca/n1/pub/11-626-x/11-626-x2019015-eng.htm (accessed 20 November).

**Statistics Canada** (2023) *Police-reported hate crime, 2021*. Available at: https://www150.statcan.gc.ca/n1/daily-quotidien/230322/dq230322a-eng.htm (accessed 20 April).

**Steen J, Loeys T, Moerkerke B and Vansteelandt S** (2017) medflex: An R Package for Flexible Mediation Analysis using Natural Effect Models. *Journal of Statistical Software* 76(11), 1 - 46. https://doi.org/10.18637/jss.v076.i11.

**Sudbury.com Staff** (2021) *Perennial Sudbury candidate found guilty of hate speech is running for office once again*. Available at: https://www.sudbury.com/local-news/perennial-sudbury-candidate-found-guilty-of-hate-speech-is-running-for-office-once-again-4271445 (accessed 10 April).

**Tchetgen Tchetgen EJ** (2014) A Note on formulae for causal mediation analysis in an odds ratio context. *Epidemiologic Methods* 2(1): 21-31.

**Treisman R** (2021) *After two failed attempts, Canada bans conversion therapy*. Available at: https://www.npr.org/2021/12/09/1062720266/canada-bans-conversion-therapy (accessed 12 April).

**UNAIDS** (2022) *Global Partnership for Action to Eliminate All Forms of HIV-related Stigma and Discrimination*. Available at: https://www.unaids.org/sites/default/files/media_asset/global-partnership-hiv-stigma-discrimination_en.pdf (accessed 1 June).

**Vaishnavi S, Connor K and Davidson JR** (2007) An abbreviated version of the Connor-Davidson Resilience Scale (CD-RISC), the CD-RISC2: psychometric properties and applications in psychopharmacological trials. *Psychiatry Research* 152(2-3): 293-297.

**VanderWeele TJ** (2013) A three-way decomposition of a total effect into direct, indirect, and interactive effects. *Epidemiology* 24(2): 224-232.

**VanderWeele TJ** (2016) Mediation Analysis: A Practitioner's Guide. *Annual Review of Public Health* 37: 17-32.

**VanderWeele TJ and Robinson WR** (2014) On the causal interpretation of race in regressions adjusting for confounding and mediating variables. *Epidemiology* 25(4): 473-484.

**Vansteelandt S, Bekaert M and Lange T** (2012) Imputation Strategies for the Estimation of Natural Direct and Indirect Effects. *Epidemiologic Methods* 1(1): 131-158.

**Waite S, Ecker J and Ross LE** (2019) A systematic review and thematic synthesis of Canada's LGBTQ2S+ employment, labour market and earnings literature. *PLoS One* 14(10): e0223372.

**Westreich D and Greenland S** (2013) The Table 2 Fallacy: Presenting and Interpreting Confounder and Modifier Coefficients. *American Journal of Epidemiology* 177(4): 292-298.

**Willems S, Schat A, van Noorden MS and Fiocco M** (2018) Correcting for dependent censoring in routine outcome monitoring data by applying the inverse probability censoring weighted estimator. *Statistical Methods in Medical Research* 27(2), 323-335. https://doi.org/10.1177/0962280216628900.

**Yu A** (2019) *The enduring legacy of Canada’s racist head tax on Chinese-Canadians*. Available at: https://macleans.ca/society/the-enduring-legacy-of-canadas-racist-head-tax-on-chinese-canadians/#:~:text=On%20June%2022%2C%202006%2C%20Stephen,to%20the%20country%20until%201947 (accessed 5 May).

**Zussman R** (2021) *Horgan ‘deeply’ troubled by 717% increase in anti-Asian hate crimes in Vancouver*. Available at: https://globalnews.ca/news/7647135/horgan-bc-presser-feb-18/ (accessed 28 March).
